# Unmet Medical Needs in Chronic, Non-communicable Inflammatory Skin Diseases

**DOI:** 10.3389/fmed.2022.875492

**Published:** 2022-06-09

**Authors:** Hideyuki Ujiie, David Rosmarin, Michael P. Schön, Sonja Ständer, Katharina Boch, Martin Metz, Marcus Maurer, Diamant Thaci, Enno Schmidt, Connor Cole, Kyle T. Amber, Dario Didona, Michael Hertl, Andreas Recke, Hanna Graßhoff, Alexander Hackel, Anja Schumann, Gabriela Riemekasten, Katja Bieber, Gant Sprow, Joshua Dan, Detlef Zillikens, Tanya Sezin, Angela M. Christiano, Kerstin Wolk, Robert Sabat, Khalaf Kridin, Victoria P. Werth, Ralf J. Ludwig

**Affiliations:** ^1^Department of Dermatology, Faculty of Medicine and Graduate School of Medicine, Hokkaido University, Sapporo, Japan; ^2^Department of Dermatology, Tufts Medical Center, Boston, MA, United States; ^3^Department of Dermatology, Venereology and Allergology, University Medical Center Göttingen, Göttingen, Germany; ^4^Lower Saxony Institute of Occupational Dermatology, University Medical Center Göttingen, Göttingen, Germany; ^5^Center for Chronic Pruritus, Department of Dermatology, University Hospital Muenster, Muenster, Germany; ^6^Department of Dermatology, University of Lübeck, Lübeck, Germany; ^7^Institute for Allergology, Charité—Universitätsmedizin Berlin, Corporate Member of Freie Universität Berlin and Humboldt-Universität zu Berlin, Berlin, Germany; ^8^Fraunhofer Institute for Translational Medicine and Pharmacology (ITMP), Allergology and Immunology, Berlin, Germany; ^9^Institute and Comprehensive Center for Inflammation Medicine, University of Lübeck, Lübeck, Germany; ^10^Lübeck Institute of Experimental Dermatology and Center for Research on Inflammation of the Skin, University of Lübeck, Lübeck, Germany; ^11^Division of Dermatology, Rush University Medical Center, Chicago, IL, United States; ^12^Department of Internal Medicine, Rush University Medical Center, Chicago, IL, United States; ^13^Department of Dermatology and Allergology, Philipps-Universität, Marburg, Germany; ^14^Department of Rheumatology and Clinical Immunology, University of Lübeck, Lübeck, Germany; ^15^Department of Dermatology, Perelman School of Medicine, University of Pennsylvania, Philadelphia, PA, United States; ^16^Corporal Michael J. Crescenz Veterans Affairs Medical Center, Philadelphia, PA, United States; ^17^Department of Dermatology, Columbia University Medical Center, New York, NY, United States; ^18^Psoriasis Research and Treatment Centre, Charité—Universitätsmedizin Berlin, Berlin, Germany; ^19^Interdisciplinary Group Molecular Immunopathology, Dermatology/Medical Immunology, Charité—Universitätsmedizin Berlin, Berlin, Germany; ^20^Azrieli Faculty of Medicine, Bar-Ilan University, Safed, Israel

**Keywords:** medical need, skin, inflammation, atopic dermatitis, psoriasis, alopecia areata, chronic spontaneous urticaria, hidradenitis suppurativa

## Abstract

An estimated 20–25% of the population is affected by chronic, non-communicable inflammatory skin diseases. Chronic skin inflammation has many causes. Among the most frequent chronic inflammatory skin diseases are atopic dermatitis, psoriasis, urticaria, lichen planus, and hidradenitis suppurativa, driven by a complex interplay of genetics and environmental factors. Autoimmunity is another important cause of chronic skin inflammation. The autoimmune response may be mainly T cell driven, such as in alopecia areata or vitiligo, or B cell driven in chronic spontaneous urticaria, pemphigus and pemphigoid diseases. Rare causes of chronic skin inflammation are autoinflammatory diseases, or rheumatic diseases, such as cutaneous lupus erythematosus or dermatomyositis. Whilst we have seen a significant improvement in diagnosis and treatment, several challenges remain. Especially for rarer causes of chronic skin inflammation, early diagnosis is often missed because of low awareness and lack of diagnostics. Systemic immunosuppression is the treatment of choice for almost all of these diseases. Adverse events due to immunosuppression, insufficient therapeutic responses and relapses remain a challenge. For atopic dermatitis and psoriasis, a broad spectrum of innovative treatments has been developed. However, treatment responses cannot be predicted so far. Hence, development of (bio)markers allowing selection of specific medications for individual patients is needed. Given the encouraging developments during the past years, we envision that many of these challenges in the diagnosis and treatment of chronic inflammatory skin diseases will be thoroughly addressed in the future.

## Chronic, Non-Communicable Inflammatory Skin Diseases

Chronic, non-communicable skin inflammation can be caused by many different diseases. Herein, we categorized these into (i) chronic inflammatory diseases (atopic dermatitis, psoriasis, lichen planus, chronic prurigo, and hidradenitis suppurativa), (ii) autoimmune diseases (alopecia areata, vitiligo, chronic spontaneous urticaria, pemphigus, bullous pemphigoid, mucous membrane pemphigoid, and epidermolysis bullosa acquisita), (iii) autoinflammatory diseases (cryopyrin-associated periodic syndrome and Schnitzler's syndrome), and (iv) rheumatic diseases (cutaneous lupus erythematosus, dermatomyositis, and systemic sclerosis). This categorization is based on the main driving pathomechanism(s) of each disease. However, a clear classification of the pathologic driver is challenging as in lichen planus and psoriasis autoreactive T- and B- cells potentially contribute to disease pathogenesis (1, 2). This classification is also expected to change over time, as it will need to adopt and consider new data on disease pathogenesis. Alternatively, to the here used classification, chronic inflammatory skin diseases may be categorized based on key driving molecules. For example, Janus kinases (JAK) in atopic dermatitis, alopecia areata, vitiligo, and cutaneous lupus erythematosus. Furthermore, as detailed below, for many chronic skin inflammatory diseases the clinical presentation varies greatly even within the same disease, as with psoriasis or bullous pemphigoid (3, 4). With the emerge of multidimensional datasets, it has been proposed to classify inflammatory skin diseases based on molecular patterns (5). The increasing understanding of (molecular) disease pathogenesis and availability of appropriate biomarkers for their identification, we expect a more complex, but more tailored categorization of molecular disease pathogenesis is leading to the emergence of potential biomarkers, and a more categorization of chronic, non-communicable skin inflammatory diseases. These diseases are a major medical burden because of their high and, in many cases, increasing prevalence (Table 1), diagnostic challenges, lack of curative treatments, co-morbidity, as well as significant economic impact. We here selected 17 chronic, non-communicable skin inflammatory diseases that collectively affect 15–20% of the population (Table 1). For each disease, the current diagnostic and therapeutic challenges are outlined. Furthermore, a perspective is given on how these challenges may be met in the future.

**Table 1:** Epidemiology of selected chronic inflammatory skin diseases.

**Disease**	**Prevalence rate**	**Sex distribution**	**Ethnic/geographic predisposition**	**Notable trends**	**References**
Atopic dermatitis	10–30% in children and 2–10% in adults	Almost equal sex distribution	Higher in high-income countries	Two- to three-fold increase over the past several decades	(6–8)
Psoriasis	2–3%	Equally prevalent in both sexes	Most common in populations of northern Europe and least common in eastern Asia	An apparent upward trend is observed in several countries	(9–11)
Prurigo nodularis	0.1%	Higher among females	None	Increasing incidence over time	(12, 13)
Lichen planus	0.2–1.3%	Equally prevalent in both sexes	• CLP: equally prevalent in both sexes • MLP: more frequent in the female population • LPP: more frequent in the female population	NA	(14, 15)
Hidradenitis suppurativa	0.1–1.3%	Overall almost equal distribution, but varies between races	Higher in African Americans	NA	(16–18)
Alopecia areata	2%	Slightly higher among females	Higher in African American and Hispanics	The incidence is increasing over time	(19)
Vitiligo	0.2–1.8%	Higher among females	Higher prevalence in African nations	Constant or decreasing frequency in the past decades	(20)
Chronic spontaneous urticaria	0.1–1.4%	Slightly higher among females	Higher prevalence in Asian nations	Increasing incidence over time	(21)
Pemphigus	Orphan	Higher among females	Higher in Ashkenazi Jewish and Mediterranean population	Inconsistent findings	(22, 23)
Bullous pemphigoid	Orphan	Higher among females	None	1.9- to 4.3-fold rise over the past two decades	(24)
Mucous membrane pemphigoid	Orphan	Higher among females	None	NA	(23)
Epidermolysis bullosa acquisita	Orphan	Equally prevalent in both sexes	HLA-DR2 and HLA-DRB1*15:03-associated susceptibility among Africans	NA	(23, 25, 26)
Cryopyrin-Associated periodic syndrome	Orphan	Equally prevalent in both sexes	None	NA	(27, 28)
Schnitzler's syndrome	Orphan	Higher among males	None	NA	(29, 30)
Cutaneous lupus erythematosus	Orphan	Higher among females	Higher in Māori/Pacific population	NA	(31, 32)
Dermatomyositis	Orphan	Higher among females	Higher among Africans and Hispanics	Increasing incidence over time	(33, 34)
Systemic sclerosis	Orphan	Higher among females	Higher among Africans and Hispanics	Increasing incidence over time	(34, 35)

*Orphan, Disease prevalence 5/10,000 or less; NA, not applicable*.

## Chronic Inflammatory Skin Diseases

### Atopic Dermatitis

Atopic Dermatitis (AD) or atopic eczema is a common, chronic, relapsing inflammatory disease, affecting up to 30% of the pediatric population and 2–10% of adults (36). While most commonly symptoms start in the first 5 years of life, it is now recognized that onset can occur at any age. There can be a significant effect on patient's quality of life and sleep due to itch and pain (37). There are also significant effects on patients' mental health with higher incidence of depression and suicide (38). The high burden of disease can interfere with work productivity, not only from the baseline disease but particularly from flares (39). Patient also have many out-of-pocket costs, including cleaning products, clothing, moisturizers, and other expenses (40). In AD, there is an interplay between barrier dysfunction, immune dysregulation, and the microbiome (41). Both genetics and environmental factors play a role in the pathogenesis (42). In AD, the stratum corneum, composed of the terminally differentiated enucleated keratinocytes called corneocytes, is often compromised. Among European Caucasians, filaggrin mutations are associated with early-onset and severe AD (43). Filaggrin is broken down into compounds that constitute natural moisturizing factor which is important for appropriate hydration, desquamation, plasticity, acidity, and the commensal microbiome (44, 45). Patients with AD have a higher burden of Staphylococcus aureus which contributes to the inflammation (46). As allergens penetrate the defective skin barrier in AD, pro-inflammatory cytokines are released. While a type 2 immune response with elevated levels of IL-4 and IL-13 predominate in the acute phase, chronically, a mixed response of Th1, Th17, and Th22 immune cells is observed (47). IL-31 is particularly implicated in pruritus (48).

#### Diagnosis

**Figure 1:**
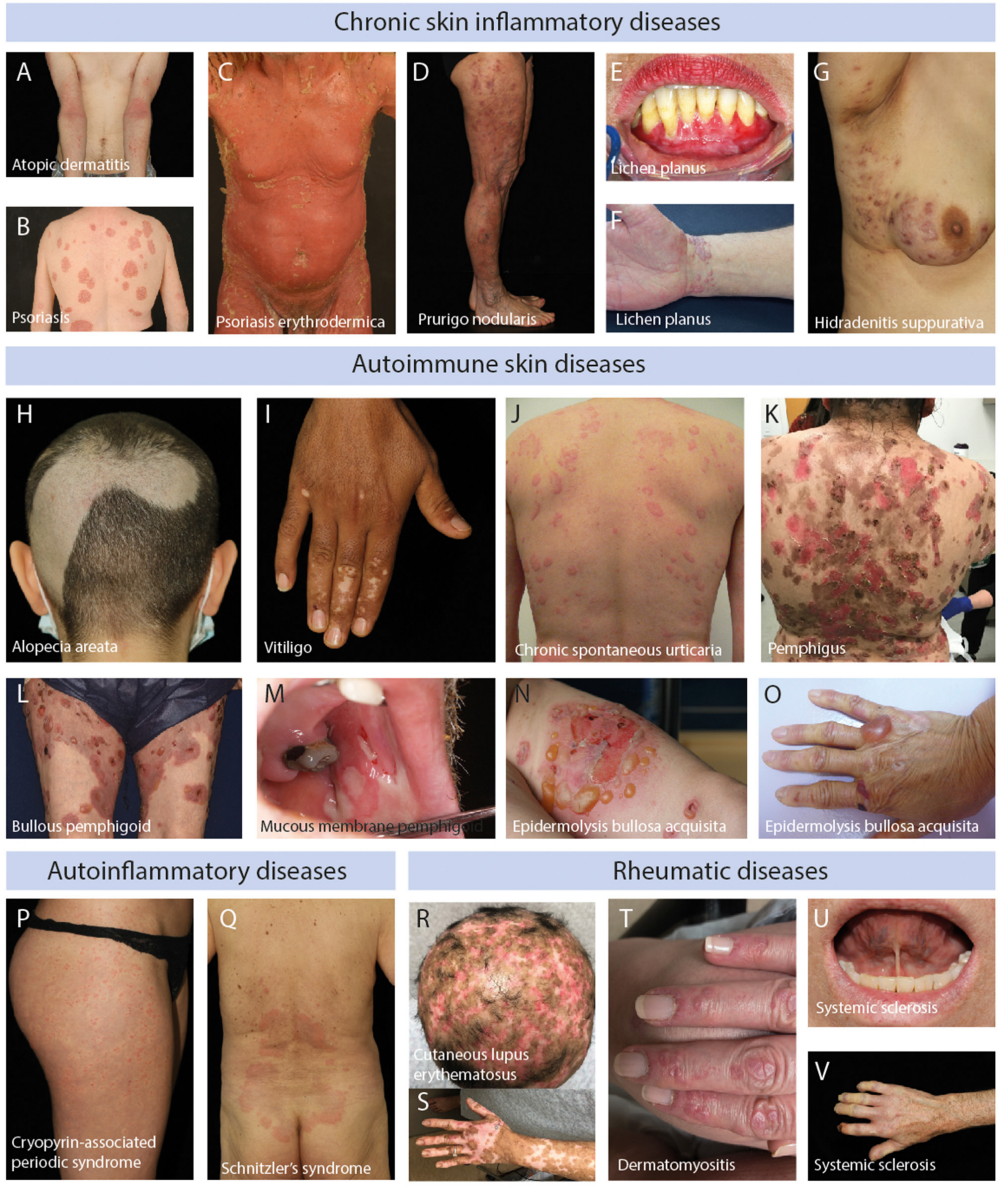
Clinical images of patients with chronic skin inflammatory diseases. **(A)** Blurry erythema and lichenification at the inside the bend of the elbows and arms of a patient with atopic dermatitis. **(B)** Sharply demarked, scaling, erythematous plaques at the back of a patient with psoriasis vulgaris. **(C)** Generalized erythema and scaling in a patient with psoriasis. **(D)** Erythematous nodules, partially excoriated, in a patient with prurigo nodularis. **(E)** Erosions of the lower gums in a patient with mucosal lichen planus. **(F)** Polygonal, scaling, reddish-violet plaques at the wrist of a patient with cutaneous lichen planus. **(G)** Scaring, nodules and pustules located at the sub-axillary region of a patient with hidradenitis suppurativa. **(H)** Sharply demarked hair loss at the back of the head in a patient with alopecia areata. **(I)** Sharpley demarked white maculae at the hands of a patient with vitiligo. **(J)** Wheals at the back of a patient with chronic spontaneous urticaria. **(K)** Brown macules and erosions at the back of a patient with muco-cutaneous pemphigus vulgaris. **(L)** Tense blisters on erythematous skin on the legs of a patient with bullous pemphigoid. **(M)** Oral erosions in a patient with mucous membrane pemphigoid. **(N)** Tense blisters on erythema on the arm of a patient with inflammatory/non-mechano-bullous epidermolysis bullosa acquisita. **(O)** Tense blister and scaring on the hand of a patient with predominant mechano-bullous epidermolysis bullosa acquisita. **(P)** Wheals at the leg of a patient with cryopyrin-associated periodic syndrome. **(Q)** Urticarial exanthema at the lower back of a patient with Schnitzler's syndrome. **(R)** Alopecia and erythema at the head of a patient with cutaneous lupus erythematosus. **(S)** Erythema and depigmentation at the arm of a patient with cutaneous lupus erythematosus. **(T)** Gottron papules in a patient with dermatomyositis. **(U)** Shortening of the sublingual frenulum in a patient with systemic sclerosis. **(V)** Raynaud's phenomenon (anemic color of the fingers) and necrosis of the index finger in a patient with systemic sclerosis.

AD is usually diagnosed based on clinical experience (Figure 1A). There are diagnostic criteria, but no simple test for definitive diagnosis (49). When patients manifest in atypical locations, develop lesions later in life, have uncommon morphologies or other overlying skin diseases the diagnosis can be challenging. AD is heterogenous and can show racial variation (50). Asians may manifest with well-demarcated lesions and skin of color patients may have increased xerosis, follicular eczema, and post-inflammatory pigmentation changes (51). Allergic contact dermatitis may overly AD, so patch testing should be considered in those with recalcitrant atopic dermatitis. AD is notably associated with other atopic disorders such as asthma, allergic rhinitis, and food allergies. There also has been an association with obesity, malignancy, and cardiovascular disease (52–54). For a precision medicine approach, validated and reliable biomarkers are needed to individually tailor treatment (55).

#### Treatment

Treatment is mainly aimed at restoring the skin barrier and modulating the abnormal immune response. Education on skin hygiene strategies is important for all patients, ideally with written action plans. There is uncertainty as to ideal bathing recommendations as it may improve skin hydration and provide some symptom relief while use of detergents may have a dehydrating effect. Emollients are a cornerstone of treatment and can lead to a decrease in the amount of prescription topical agents needed to treat AD (56). However, it is not known the optimal amount or frequency of emollient application. Additionally, there are some moisturizers that may irritate the skin of individual patients. Besides emollients, topical agents including corticosteroids are first-line therapy. Non-steroidal options such as topical calcineurin inhibitors (TCIs) are useful for areas of sensitive skin such as face, neck, and genitals. Calcineurin inhibitors can also be used as maintenance twice a week to reduce the frequency and severity of flares (57). While there was initial concern regarding the use of TCIs and the risk of malignancy, post-marketing research has been reassuring as to the safety of these treatments (58). In patients who fail topical treatment, phototherapy, oral immunosuppressants, and targeted biologics are indicated. In particular, the anti-IL4 receptor alpha inhibitor dupilumab has changed the way we treat AD in both pediatric and adult patient. While sedating antihistamines for short-term use can assist with sleep disturbance caused by pruritus, there is a lack of evidence to support the use of non-sedating and sedating antihistamines for generalized, extended use. Due to cumulative side effects, oral corticosteroids should be avoided in the long-term and in children. There are many exciting new mechanisms of action in development (or very recently approved) to treat atopic dermatitis including aryl hydrocarbon receptor agonists, commensal bacteria, JAK inhibitors (JAKi), and new biologics that target IL-13, IL-31, IL-33, and OX-40 (59, 60).

#### Perspectives

Atopic dermatitis is one of the most common inflammatory skin disorders and there are still multiple unmet needs and educational gaps. Instructing patients and caregivers regarding skin hygiene with liberal use of emollients is essential for all. Additionally reassuring fears of corticosteroids is an important task of providers. There is no generally accepted goal of treatment, so currently plans are individualized for patients with a need for biomarkers and research into personalized medicine. Adherence to therapy remains a long-term challenge as care of atopic dermatitis can be quite time consuming and costly. There are an increasing number of therapeutic options that are being developed due to our improved understanding about the pathogenesis of AD and with it, improved hope at helping more patients who suffer from atopic dermatitis.

### Psoriasis

Over the past three decades, psoriasis has become a model disease for the study of chronic inflammatory diseases. Several new drugs have been and are being developed first for psoriasis and then extended to other indications (61). Central to our current understanding of the pathogenesis of psoriasis is a close interaction between components of the innate and adaptive immune systems (62, 63). For example, the former branch is represented by macrophages, neutrophilic granulocytes, and (plasmacytoid) dendritic cells; the latter by T lymphocytes, primarily Th17 cells. Communication between these immune cells is mediated by various cytokines including TNFα, IL-17, and IL-23 which have become targets of multiple biologic therapies (64, 65).

#### Diagnosis

Psoriasis is diagnosed based on history and clinical presentation—only rarely a biopsy is needed to confirm the diagnosis. Comorbidity, especially psoriatic arthritis should be excluded at diagnosis and during follow-up (62). However, psoriatic disease is not a uniform disease entity (Figures 1B,C). Although current drugs were developed and approved for the so-called chronic plaque psoriasis, we encounter psoriasis in the clinic as a spectrum ranging from acute exanthematous to chronic stable, from classically scaly and sharply demarcated plaques to highly inflammatory, pustular or erythrodermic forms, or from forms restricted to a few predilection sites to generalized or inverse forms. Specific underlying genetic patterns have now been identified for some of these manifestations; e.g., in *IL36RN* (3, 66). The involvement of other organ systems (comorbidity) and provoking factors in psoriasis as a systemic disease also influence the disease process. To account for the increased inflammation throughout the organism with (possible) systemic impairment of several other organ systems, we now tend to refer to it as psoriatic disease.

#### Treatment

There are well-defined guidelines for the treatment of psoriasis (67, 68). The following drugs are FDA or EMA approved for psoriasis and/or psoriatic arthritis: TNFα inhibitors etanercept, infliximab, adalimumab, certolizumab pegol, and golimumab; IL-17a inhibitors secukinumab and ixekizumab; IL-17A and IL-17F dual inhibitor bimekizumab; IL-17 receptor A/C inhibitor brodalumab; IL-12 and IL-23 inhibitor, ustekinumab; and IL-23 inhibitors guselkumab, tildrakizumab, and risankizumab (69–72). Very recent developments also include mirikizumab and netakimab (73). The development of specific therapeutics against essential cytokines in the IL-23/IL-17 axis is a good illustration of how basic and translational immunological research has led to the development of highly potent drugs that can effectively and safely treat most patients with at least moderate psoriasis. These therapies have been and continue to be included in current guidelines (67, 68, 74, 75). In addition, small-molecule drugs have been and are being developed, such as apremilast against phosphodiesterase 4 (PDE4), deucravacitinib against tyrosine kinase 2 (Tyk2) and piclidenoson, a Gi protein-associated A3 adenosine receptor agonist (73). Compared to the era before biologics, the impact has been so great that it is fair to label these treatments revolutionary. Thus, we are now in a fairly comfortable position with regard to the treatment psoriatic disease: there are now numerous effective and well-tolerated preparations available and due to competitive pressure and the increasing availability of biosimilars, the price will (hopefully) come down in such a way that more and more patients can be treated with these systemic therapeutics. Still, there are still unmet medical needs for psoriasis that related to diagnostics and treatment. Specifically, in terms of personalized and precision medicine, however, there is definitely still a need for development here, since by no means do all of our patients meet the “standard” of chronic plaque psoriasis, which is usually considered in registration studies. On this basis, later extensions of indications are also conceivable for diseases that have similar pathogenetic features and for which, due to their relative rarity, large prospective clinical trials are usually not conducted (76). The categorization and characterization of inflammatory and autoimmunity patterns, which is already under development, may help in this regard (77). There is also not yet enough data on combination therapies for psoriasis (78). In particular, combinations of modern and conventional therapies could in some cases increase effectiveness and reduce costs. Similar considerations apply to individualized dosing regimens and terminations of therapies.

#### Perspectives

It is thus clear that the pathogenesis of psoriasis is complex. Increasingly, it is becoming clear that the overall pattern of inflammatory mediators and cells, which may well shift over the course of the “disease career,” is ultimately responsible for the individual form of manifestation. Therefore, it is reasonable to strive for a more detailed understanding of these inflammatory patterns and the factors regulating them on a “holistic” level. Hence, the establishment and clinical validation of biomarkers and molecular genetic patterns could enable predictions of response or loss of efficacy of specific therapies in individual patients (79). If successful, patients could quickly and sustainably receive the most appropriate therapy for them and we would avoid unnecessary delays, side effects and costs. This is only beginning to happen and should improve over time (80). Regarding therapeutics, development is proceeding in two major directions: On the one hand, further mediators are being inhibited in a targeted manner and with more refined methods and reagents. The most recently approved example is an antibody that blocks the effects of both IL-17A and IL-17F (bimekizumab) (81). The clinical effectiveness of this approach is convincingly good. The development was prompted by scientific findings that although IL-17A, which was initially considered, has a much higher affinity at the receptor, that the homologous IL-17F isoform is present in much greater amounts in psoriatic skin. In addition, IL-17A and IL-17 F may also act as heterodimers at the receptor (82). Other interesting developments also include mediators that have been primarily attributed to more innate immune mechanisms that may factor more strongly in therapeutic considerations and developments. A good example of this is the development of IL-36 antagonists or blockade of other members of the IL-1 family (83), which are proving to be promising in pustular and highly inflammatory forms of psoriasis (84). Besides biologics, small-molecules which are orally available and able to penetrate cells are being pursued. They inhibit central signaling pathways in pathogenetically relevant cell types. Due to their small molecular size, these substances are in principle also suitable for topical application. In contrast to the older preparations such as methotrexate, retinoids, or fumaric acid esters, whose effects are quite broad and where mechanisms have not yet been completely clarified, the newer preparations have a more selective effect. Despite supposed selectivity, however, the effect is sometimes more pleiotropic and there are more off-target effects than with biologics. The first compound approved for the treatment of psoriasis in this group is the PDE4 inhibitor apremilast. In late stages of clinical development is the TYK2 inhibitor deucravacitinib, which in clinical trials offers quite convincing clinical effects with a good safety profile. The background for the latter development is the recognition that many mediators, for example type I interferons or IL-23, mediate their inflammatory signals via Janus kinases (JAK, to which TYK2 also belongs) (85, 86). Thus, by blocking these signaling molecules, an (indirect) inhibition of inflammatory mechanisms can be achieved.

To address most of these challenges, it is likely that proteogenomic approaches will have to be expanded, implemented and/or developed in conjunction with sophisticated immunological-functional studies. These will need to be supported by comprehensive “real world” data, for example from registry studies, to capture the full actual spectrum of psoriatic disease. Only in this way will it be possible to stratify the heterogeneous population of patients with psoriatic disease even more precisely in cross-section and to characterize it down to the level of the individual. It will also enable a more accurate longitudinal characterization of the disease over its lifelong course. The ultimate goal must be to find and apply the most effective, and at the same time best tolerated therapy for each individual patient at any point in their “disease career,” sometimes in a quite variable manner (87).

### Prurigo Nodularis

Prurigo nodularis (PN) belongs to the spectrum of chronic prurigo and it is the dominant phenotype for 70% of patients with chronic prurigo (13, 88, 89). Chronic prurigo is a persistent and burdensome neuroinflammatory dermatosis associated with severe itching, permanent scratching behavior and diverse comorbidities. Among the comorbidities, for example atopic predisposition, atopic dermatitis, bullous pemphigoid, lichen planus, chronic kidney disease, hepatobiliary diseases, diabetes mellitus, chronic iron deficiency, HIV, and solid tumors have been reported to be causally related to PN (90). However, the role of these comorbidities as etiological factor in PN is still debated and needs additional studies. PN affects both sexes, all races and all ages with a preference for females above age of 60 years (90). Children may be affected, but this is very rare. Epidemiological studies are infrequent and report different prevalences depending on the method and population included (13, 91, 92). For example, Poland reported an estimated prevalence of 6.5/100,000, USA of 36.7 to 43.9/100,000 and Germany up to 100/100,000. However, all studies seem to argue for the fact that PN is not really a rare disease. Currently, neuroimmune mechanisms are considered the dominant mechanism underlying PN (93). In PN skin, different helper T cell phenotypes have been identified including Th1, Th2, Th17, and Th22 cells. Especially Th2 cytokines such as interleukin (IL)-4 and IL-31 are abundantly present in PN skin. Cutaneous sensory C- and Ad- nerve fibers express corresponding IL receptors. This enables a close neuroimmune communication with continuous stimulation of nerve fibers, their release of neuropeptides and induction of itch. Interestingly, IL31 has a prominent role not only enhancing the inflammation, but also leading to epidermal hyperplasia and fibrosis formation of the dermal collagen tissue. In addition to IL-31, also upregulated periostin might promote fibrosis formation by releasing IL31 from various immune cells (94, 95) from various immune cells. Fibrosis is a prominent feature in PN and distinguishes the disease histologically from atopic dermatitis. In addition, IL-4 plays a major role in fostering neuroimmune communication and neuronal hypersensitivity *via* the IL-4Rα/JAK pathway. Recent studies suggested that nerve fiber dysfunction and structural neuroanatomical changes are present in PN skin which are induced by scratching and maintained by inflammation (96, 97).

#### Diagnosis

The diagnosis of PN is made clinically (98). PN is characterized by symmetrically distributed nodules; papules, and plaques may occur (Figure 1D). The severity of PN can range from some single nodules to several hundreds. All lesions are itchy and subject to scratching, formation of excoriations and bleeding. Lesions are found mainly on the extensor surfaces of the extremities and trunk with a typical butterfly sign at the back (lack of lesions at the central back which can be explained by the patient's inability to scratch these skin areas). Palms, soles and the face are rarely affected. PN can be documented by a PN-specific, validated investigator global assessment (99). The validated investigator questionnaire Prurigo Activity and Severity Score (PAS) assesses several parameters of the disease such as type, number, and distribution of pruriginous lesions, proportion of PN lesions with excoriations and proportion of healed lesions (100). Itch intensity is monitored best with validated instruments such as the numerical rating scale (NRS) for example as it is also done in other types of pruritus (101). The health-related quality of life can be documented either by Dermatology Life Quality Index (DLQI) or the itch-specific ItchyQol (102).

#### Treatment

The first international guideline on diagnostic and therapy of PN recommends a laddered approach to treat PN (98). The first two steps comprise topical and intralesional corticosteroids, topical calcineurin inhibitors, capsaicin, systemic antihistamines and UV-phototherapy. In the third step, either neuronal therapies or immunosuppressants are advised such as gabapentinoids, antidepressants as well as cyclosporine and azathioprine. The clinical findings (inflamed vs. non-inflamed nodules) and quality of itch (itch with pain, burning and stinging) may guide to the right therapy.

#### Perspectives

Novel, effective, safe and approved therapies are urgently needed as patients are in general dissatisfied with the currently medical care (103). Currently, opioid modulators and investigational substances (dupilumab, nemolizumab) are recommended as off-label treatments in refractory cases (104, 105). For some of these substances, clinical trials are currently being conducted. For example, opioid modulators as well as IL4 and IL31 receptor antibodies are in current pharmaceutical development. IL31 signals through a heterodimeric receptor complex consisting of IL-31 receptor α (IL-31RA) and oncostatin M receptor β (OSMRβ). Novel substances target each of the two receptor components and are in phase II and phase III stage of development.

### Lichen Planus

Lichen planus (LP) is a chronic immune-mediated disease which affects skin, mucosa and skin appendages. LP is the prototype of a lichenoid dermatosis which is characterized by a dense dermal T cellular and macrophage-rich infiltrate. LP is a common disease with an incidence in the general population is up to 1.27%. While LP is most common in the third and sixth decade, it may occur at any age. Mucosal LP (MLP) shows a prevalence of 0.89% and it is more commonly diagnosed in the female population. Involvement of the scalp is also more often reported in female patients, with a sex ratio close to 5:1. Clinically, we recognize three major subtypes of LP: cutaneous LP (CLP), MLP, and LP of the scalp, classically called lichen planopilaris (LPP) (14, 106, 107). CLP is classically characterized by violaceous, polygonal, slightly scaling and extremely pruriginous flat papules, which affect mostly the extremities (Figures 1E,F). Typically, CLP lesions show Wickham striae, whitish net-like lines that represent the clinical expression of the histologically seen epidermal hypergranulosis. Furthermore, several variants of CLP have been reported in the literature, including annular LP, atrophic LP, and LP verrucosus. A rare entity is represented by LP pemphigoides, which is clinically characterized by papules and blisters and serologically by the detection of IgG autoantibodies against BP180 and BP230 (108–110). MLP affects most frequently the oral mucosa. It has been more often described in female patients in the fourth decade (107). Clinically, MLP of the oral cavity is characterized by Wickham striae, erythematous macules, and, in some aggressive cases, by ulcerations (Figure 1E). A concomitant genital involvement has been reported in every second female patient affected by oral MLP (106). LP can involve other mucosal sites, including ocular, laryngeal, and esophageal mucosa. In the last case, the presence of dysphagia or odynophagia has been frequently reported, in 80 and 30% of cases, respectively (111). LPP is clinically characterized by red papules or plaques and perifollicular erythema. The chronic inflammation leads to destruction of hair follicles and to development of scarring alopecia. Patients affected by LPP may experience itching, burning of the scalp, and hair fragility. LPP requires an intensive and long-lasting therapy because of the characteristic refractory course of the disease. A variety of drugs may trigger LP, including antibiotics (e.g., dapsone and tetracycline), antifungal, and antimalarial drugs. Therefore, a detailed pharmacologic history is mandatory. The typical histological feature of LP is a band-like lymphohistiocytic infiltrate at the dermal-epidermal junction and in the upper dermis. Furthermore, hypergranulosis, irregular hyperplasia of the rete ridges with a classical saw-toothed pattern, and basilar vacuolar degeneration have been typically reported. Apoptosis of epidermal keratinocytes leads to the development of Civatte bodies, described as rounded, homogenous, eosinophilic cellular deposits in the upper dermis that can be identified by PAS staining and direct immunofluorescence microscopy (112).

#### Diagnosis

The diagnosis should be performed according to the clinical and histopathological features. In addition, several differential diagnoses should be excluded, such as lichenoid drug eruptions, lichen planus pemphigoides, graft-vs. host disease, granuloma annulare, oral candidiasis, and oral leukoplakia (113). However, diagnosis is often delayed because of the highly variable clinical appearance and inconsistent histopathological findings in LP (114).

#### Treatment

At times, LP may pose a therapeutic challenge. Indeed, some clinical variants are characterized by a refractory course, especially LPP, ulcerative oral LP and genital LP (14, 109). In CLP topical steroid treatments usually in combination with UVB or PUVA phototherapy are recommended. In recalcitrant cases, oral prednisone or oral retinoids may be useful (115). Oral LP can be initially treated with topical potent corticosteroids (e.g., clobetasol propionate 0.05%). Intralesional injection of corticosteroids can be useful in ulcerative oral LP. In addition, an off-label therapy with topical application of pimecrolimus or tacrolimus can be used. In case of severe involvement of the oral mucosa, several systemic therapies have been tried, including systemic corticosteroids, azathioprine, methotrexate, and retinoids (115, 116). In LPP an early and rapid control of inflammation is of pivotal importance to prevent the development of scaring alopecia. Topically, potent corticosteroids can be used in moderate cases (115). Alternatively, treatment with topical calcineurin inhibitors or with topical JAKi (e.g., tofacitinib) have been shown to be effective (109, 117). In more aggressive cases, a concomitant treatment with systemic corticosteroids is recommended. Alternatively, hydroxychloroquine or methotrexate can be used as second-line treatment. In recalcitrant cases, mycophenolate mofetil or cyclosporine A can be used as off-label treatment (118).

#### Perspectives

Recently, the use of anti-IL-17, anti-IL-12/IL-23, and anti-IL-23 monoclonal antibodies was reported to lead to an improvement of oral ulcerations in extremely refractory cases (109). This off-label use of these therapeutics was based on the observation of a Th1/Th17-dominated cell response in the peripheral blood of LP patients (1). To address, if this pathway is amendable to pharmacological interventions, a total of five patients with lichen planus were treated in a compassionate use trial. Of these, three received secukinumab, one patient ustekinumab and one guselkumab. In all cases, marked improvement was documented within the 12-week observation period. Of note, the clinical improvement was accompanied by a strong reduction of the Th1 and Th17/Tc17 cellular mucosal infiltrate, suggesting that IL-17-producing T cells are central to disease pathogenesis (109) At this regard, an open label, parallel, randomized, multi- center, phase II trial to evaluate the efficacy, ssafety, and tolerability of guselkumab in patients with oral LP is now ongoing (EudraCT Number: 2021-000271-36). In addition, a phase II study to evaluate the efficacy, safety, and tolerability of secukinumab 300 mg over 32 weeks in adult patients with biopsy-proven clinical variants of LP is ongoing (EudraCT number 2019-003588-24). Furthermore, JAKi have emerged as promising therapeutic agents in LP (14, 117).

### Hidradenitis Suppurativa

Patients with hidradenitis suppurativa (HS) suffer from chronic painful inflammatory skin lesions in intertriginous sites (119) (Figure 1G). The manifestation of the disease mostly occurs around the age of 25. The average prevalence rate of HS is 0.2–0.4%, with highest rates in the Caucasian (0.75%) and the African American populations (1.3%) (17). Both sexes are affected with similar frequencies (16, 18, 120). Besides skin alterations, HS patients commonly suffer from numerous systemic comorbidities such as metabolic syndrome, spondyloarthritis or spondyloarthropathy (SpA), mental depression, and inflammatory bowel disease (121–125). HS leads to profound impairment in the quality of life of affected people, which is much more pronounced than the impairment caused by other dermatoses (16, 126). Furthermore, HS is associated with patients‘ body image impairment and increased suicidal behaviors (127–129). Ischemic heart diseases as well as accidents and violence (incl. suicides) contribute to the massively shortened (~15 years) life expectancy of patients with HS (130).

#### Diagnosis

The diagnosis of HS is based solely on the physical examination and medical history (119). HS is confirmed when the following criteria are met: (i) typical skin alterations such as inflammatory nodules, abscesses, inflamed and draining tunnels (sinus tracts or fistulas), and rope-like scarring, (ii) in typical localizations such as in axillary (armpits), inguinal (groin), gluteal, perianal, and submammary (women) areas of the body, and (iii) typical occurrence, i.e., persistent (at least 6 months) or recurrent (>2 skin lesions occurring or recurring within 6 months) (119). Surprisingly, diagnosis is frequently delayed, although the diagnostic criteria are very clear (120). In Germany, the average duration between the manifestation of first symptoms and the HS diagnosis is 10 years (120). Importantly, the longer the delay of diagnosis, the more misdiagnoses, the greater the disease severity at diagnosis, and the higher the number of concomitant diseases (120). Various clinical scores are used to assess the severity of HS skin alterations. The International Hidradenitis Suppurativa Severity Score System (IHS4) is becoming increasingly important. It is a dynamic score based on the number of nodules, abscesses, and draining tunnels and allows dividing the severity of the disease into mild, moderate, severe (131). Patient-reported outcome measures, like DLQI, are also often used to assess the disease impact (132–134). Several blood biomarkers reflecting the activity of the immune system have been suggested, but none of them is currently used in everyday practice (135–137).

#### Treatment

Treatment options for HS lesions include pharmacological therapies (local and systemic) and surgical treatments (119). The choice of therapy depends on the type and severity of the skin lesions as well as the patient's expectations. Individual inflamed nodules can be treated with topical antiseptic or antibiotic ointments or creams. If there are several inflamed nodules or abscesses, local therapy is supplemented by systemic pharmacological treatment. Irreversible skin alterations such as tunnels and scars can be effectively treated by surgery. Therefore, it is highly important to prevent such alterations from occurring through timely and effective pharmacological therapy. A combination of clindamycin and rifampicin is commonly used for systemic treatment. However, surgical and conventional pharmacologic therapies of HS are not associated with long-lasting improvement of patients' quality of life (132). Furthermore, the anti-TNF-a antibody adalimumab is the only approved systemic treatment for HS so far (119). Thus, one of significant challenges in HS care is the lack of further systemic treatment options. This limitation is basically due to our limited understanding of the molecular and immunological processes underling the formation and persistence of skin alterations in HS (138). TNF-a, IL-1, 5-lipoxygenase and G-CSF are thought to have a role in HS pathogenesis, but the pathophysiology is not well-understood (139–142).

#### Perspectives

Due to the long delay in diagnosis, the enormous impairment of the quality of life caused by HS and the limited range of evidence-based therapies, patients with HS have an enormous unmet medical need. To change this situation, first and foremost the time interval between first symptoms and diagnosis must be significantly shortened. This is extremely important because of the progressive nature of the disease that over time leads to irreversible skin destruction. To this end, the patients must be pharmacologically treated as soon as the first symptoms appear. Training of doctors such as general physicians, dermatologists, surgeons, and gynecologists, as well as programs to raise/create the awareness of the disease within large parts of the society are needed. It is gratifying that many clinical trials with a focus on new systemic pharmacological treatment are currently being carried out. However, these are often without a well-founded scientific rationale and a better understanding of disease mechanisms is needed. Thus, we need extensive, well-founded translational research into the pathogenesis of the HS as the basis for the development of targeted systemic therapies for HS. A further aspect is a holistic view of the patient to include awareness of systemic inflammation evaluation, treatment of systemic comorbidities and pain, and psychological care for the patient. The last aspect is to motivate and support the patient in changing lifestyle factors that can contribute to the persistence of HS, such as smoking and obesity. Structured patient counseling that provides information about these associations, including referral to smoking cessation programs and weight loss might be helpful.

## Autoimmune Skin Diseases

### Alopecia Areata

Alopecia areata (AA) is an autoimmune skin disease that affects ~2% of the worldwide population (19). In AA T cells attack the hair follicles causing an inflammatory, non-scarring, hair loss that is typically manifested in patches as a single or multiple well-demarcated areas. Patients with the patchy form of alopecia areata (AAP) commonly exhibit hair loss on the scalp but may also present hair loss in other hair-bearing areas of the body (Figure 1H). The disease course varies greatly between AA-affected individuals in terms of disease severity, duration, and prognosis. Hence, AA patients may present patchy, diffuse, confluent, or mosaic patterns of hair loss during a single episode, or recurrent disease episodes. Additionally, while, up to 75% of the AAP patients exhibit a spontaneous regrowth of hair within a few months (143), in up to 25% of all AAP patients, the disease progresses to its more severe form, and the hair loss extends to the entire scalp (alopecia totalis; AT) or body (alopecia universalis; AU) (144). In addition to hair loss, nail abnormalities, most commonly pitting and trachyonychia, are observed in patients with AA and are more prevalent in patients with AT and AU (145).

#### Diagnosis

The diagnosis of AA is typically made based on the patient's medical history and a clinical examination that determines the location and the extent of hair loss, and differentiates AA from other potential causes of hair loss, thereby providing a more accurate prognosis and identifying a favorable line of treatment (146). The clinical examination is often supported by a positive hair pull test at the periphery of the lesion, especially in patients with active disease. Additionally, the clinical diagnosis is frequently accompanied by trichoscopy examination that is used to examine the hair follicle, hair shaft, and the surrounding skin, and establish the phase of the disease (147). Dermatoscopic findings in AA may vary depending on the specific disease phase (146). In the acute phase of AA, exclamation point hairs that are located at the border of the plaque and broken hairs that are thicker proximal to the scalp are typically observed, while in the chronic stage of AA, dystrophic hairs, uniform black dots, and yellow dots, are predominantly present. In cases with an unclear clinical presentation, clinical diagnosis is supported by a histological examination of a horizontally sectioned 4 mm scalp biopsy taken from an area of active hair loss (148). In the acute phase, a peribulbar infiltrate is observed that consists predominantly of CD4+/CD8+ T cells and Langerhans cells as well as eosinophils, mast cells, and plasma cells, in a typical “swarm of bees” pattern. In addition, pigment incontinence may also be present, due to the destruction of melanocytes in the apex of the dermal papilla. Other histological signs of AA characteristic of acute and the subacute phases include hair follicle miniaturization, a decreased anagen-to-telogen ratio, and a decreased terminal-to-vellus hair ratio (148).

#### Treatment

The first lines of therapy in most AA patients include corticosteroids and/or immunotherapy that is aimed at containing the inflammation and promote the recovery of dystrophic hair follicles. The type of treatment assigned is determined based on the age of the patient, and the extent and the severity of hair loss. The first line of therapy in AAP patients with active disease include intralesional (triamcinolone acetonide, triamcinolone hexacetonide, and hydrocortisone acetate) and topical corticosteroids (desoximetasone, betamethasone valerate, and clobetasol propionate), which show low solubility and promote maximum local anti-inflammatory actions with minimal systemic side effects (149). The adverse effects of topical and intralesional corticosteroids include folliculitis, reversible skin atrophy, telangiectasia, and hypopigmentation. In more severe cases, to contain rapidly progressing hair loss in AAP patients, systemic high-dose pulsed oral, or intravenous glucocorticoids (prednisolone) are recommended (150–152). However, one major drawback of this line of therapy includes recurrence of hair loss after therapy is discontinued (153). In AT, AU, and AAP patients with a chronic disease or in AAP patients with an active disease who fail to respond to topical or intralesional corticosteroids, topical application of contact allergens is recommended. In this line of therapy, potent contact allergens such as 1-chloro, 2, 4, dinitrobenzene (DNCB), diphenylcyclopropenone (DPCP), or squaric acid dibutyl ester (SADBE) are applied weekly onto the lesion to induce mild contact dermatitis, which *via* yet incompletely understood molecular mechanism, results in regrowth of hair (154, 155). Several side effects associated with this treatment include severe contact dermatitis, occipital or cervical lymphadenopathy, urticaria, dyschromia, and vitiligo (156, 157). Lastly, systemic glucocorticoids and systemic immunosuppressives (methotrexate, sulfasalazine, and azathioprine) can be used in patients with active AT and AU. Recently, a new class of small molecules known as JAKi were shown to be effective in AA. JAKi are especially effective in AA since they target a family of tyrosine kinases JAK1/2 and JAK1/3 that transduce cytokine-mediated signaling in T cells, which were shown to play a critical role in AA (158). Blockade of JAK1/3 and JAK 1/2 by the oral selective inhibitors, tofacitinib, and ruxolitinib, respectively, was shown to be effective in inducing regrowth of hair in AAP and AT/AU patients with active and chronic disease (159, 160). Although, no adverse side effects of these drugs were reported in AA patients, increased risk of infections and neoplasia were observed in rheumatoid arthritis patients treated with tofacitinib (161). Thus, future investigations into the potential side effects of prolonged treatment with JAKi, as well as examining the efficacy of topical JAKi formulations in AA, are required.

#### Perspectives

The heterogeneous clinical presentation, variability in the rate of spontaneous remission, and differences in disease prognosis still pose significant difficulties in assessing the efficacy of therapy in AA, making it challenging to generalize a certain line of treatment for different AA patients. Future, well-powered randomized placebo-controlled trials are required to systematically assess the efficacy of existing lines of therapy and facilitate the development of FDA-approved treatment options in AA. Large randomized placebo-controlled trials are underway for at least 3 JAKi in AA (Baracitinib, deuterated Ruxolitinib, and Ritlecitinib) with several others already approved for other inflammatory diseases (162, 163). Despite the significant improvements in our understanding of the pathophysiology of AA, future research is warranted to understand the contribution of environmental triggers to AA pathogenesis, since only a 55% concordance rate was observed in monozygotic twins, suggesting other factors contribute to disease onset (164).

### Vitiligo

Vitiligo is an autoimmune depigmenting disorder of the skin. The depigmentation results from the loss of epidermal melanocytes. Clinically presenting with well-demarcated white patches on the body, vitiligo can be cosmetically very disabling and create a psychological burden (165, 166). There has been a great advance in understanding the pathological basis due to current research. JAK kinase signaling pathways and the cytokines involved in the Th1 pathway are the focus of the upcoming vitiligo treatments, followed by antioxidant and repigmenting agents (167).

#### Diagnosis

Vitiligo is usually diagnosed clinically (168). Occasionally skin biopsy may be recommended (169). A characteristic histological hallmark is the absence of melanocytes and epidermal pigment (Figure 1I). Screening to assess potential autoimmune diseases is recommended.

#### Treatment

Therapy of vitiligo is currently unsatisfactory. Topical treatments include corticosteroid and calcineurin inhibitors (170). Phototherapy, ranging from broadband, or narrowband UVB to psoralen plus UVA, may be another option (171). In severe or treatment-refractory cases systemic treatments include mini-pulses of oral steroids, methotrexate, cyclosporin or mycophenolate mofetil. Currently, there are several drugs available, alone or combination, aiming to arrest progression and induce repigmentation of the skin. The degrees of repigmentation vary (172). Of note, there is no approved treatment for vitiligo repigmentation and current off-label therapies have limited efficacy. This emphasizes the need for better treatment options.

#### Perspectives

It is essential to increase awareness of the comorbidities associated with the disorder. The most common comorbid conditions of vitiligo are thyroid disease, diabetes mellitus, Addison's disease, pernicious anemia, rheumatoid arthritis, inflammatory bowel disease, ocular and audiological abnormalities, alopecia areata, systemic lupus erythematosus, Sjögren's syndrome, dermatomyositis, scleroderma, psoriasis, and atopic dermatitis (173). Among emerging treatments that may meet the need for safe and effective vitiligo treatments, JAK inhibitors (topical and oral) are the most promising new class of drugs currently available and act best in conjunction with phototherapy (174–177). The result from the phase III TRuE-V clinical trial program (NCT04052425 and NCT04057573), evaluating the topical JAKi ruxolitinib (Opzelura™ cream) showed a substantial repigmentation of vitiligo lesions. Hence, approval in the U.S. and Europe is expected in the upcoming months. Further treatment potential options like phosphodiesterase inhibitors (PDE4) or abatacept, a fully human fusion protein of CTLA-4 and the Fc portion of human IgG1 are sometimes used off-label. Considering the role of PD-1 ligand (PD-L1, a PD-1 agonist) and CTLA-4 in maintaining immune balance, targeting this pathway could be a therapeutic option. Furthermore, it was shown, that IL-15 acts *via* JAK STAT signaling pathways and has been recently implicated in oxidative stress mediated destruction of melanocyte. Thus, the future of vitiligo treatment may rely on the development of more specific drugs (167).

### Chronic Spontaneous Urticaria

Chronic spontaneous urticaria (CSU) is defined by the occurrence of itchy wheals, angioedema, or both for longer than 6 weeks (178). In most patients, CSU lasts for several years and then shows spontaneous remission. Because of the severe pruritus and the unpredictability of the occurrence of the signs and symptoms, most patients who are not adequately treated are severely affected in their quality of life (179). CSU is a mast cell-driven disease, and its signs and symptoms occur in response to the activation of skin mast cells and their subsequent release of histamine and other mediators. The exact underlying pathomechanisms of skin mast cell activation in CSU are not fully understood. Based on recent evidence, three subtypes of CSU have been described, type I autoimmunity (or “autoallergy”), type IIb autoimmunity (“classical autoimmunity”), and CSU due to unknown cause (180). In addition, other factors such as acute infections, certain drugs or stress modulate mast cell activation and drive exacerbations or worsening of CSU.

#### Diagnosis

In most patients, the diagnosis of CSU is straightforward, with spontaneously recurring itchy wheals, angioedema, or both, for longer than 6 weeks (Figure 1J). The current guideline on the definition, classification, diagnosis, and management of urticaria recommends a detailed patient history, physical examination (including pictures from patients) and a basic diagnostic workup consisting of a complete blood count with differential, CRP, IgG anti-TPO and total IgE (178). The questions and investigations are mainly aimed at ruling out rare differential diagnoses, for example urticaria vasculitis, autoinflammatory syndromes or bradykinin-mediated angioedema, assessing patients for underlying causes and modifying conditions, and identifying comorbid diseases and consequences of having CSU (180). Based on the answers to the respective questions, additional investigations such as histological examination of a skin biopsy or further laboratory analyses may be necessary. An important aspect of the diagnosis is the assessment of CSU activity, impact, and control. For this purpose, the urticaria activity score (UAS), the chronic urticaria quality of life questionnaire (CU-Q2oL) and the urticaria control test (UCT) should be used (178). In CSU patients with angioedema, the angioedema activity score (AAS), the angioedema quality of life questionnaire (AE-QoL), and the angioedema control test (AECT) should also be used (178).

#### Treatment

The goal of any treatment in CSU is the absence of signs and symptoms, complete disease control and a normal quality of life. To achieve this, an effective prophylactic treatment is required for all patients. The use of a 2nd generation H1-antihistamine is the recommended first-line treatment for CSU, first at standard dose and then, if needed, at up to 4-fold the standard dose (178). While 2nd generation antihistamines have proven to be a very safe long-term treatment, also at higher than standard doses (181), many patients with CSU do not achieve complete response. For those patients, the second step in the treatment algorithm is the addition of the monoclonal anti-IgE antibody omalizumab, which has been shown to be effective and safe in many H1-antihistamine refractory CSU patients (182). A significant proportion of CSU patients do not achieve complete control with omalizumab. Recent data indicate that patients with markers of type IIb autoimmune CSU, e.g., low total IgE and elevated levels of IgG anti-TPO, show slow and poor response to omalizumab treatment (180, 183). In patients who do not respond to omalizumab within 6 months of treatment (or earlier, if symptoms are unbearable), cyclosporin up to 5 mg/kg body weight is recommended in addition to antihistamines. Due to the poor safety profile, this is not possible in all patients and potential side effects should be rigorously monitored.

#### Perspectives

Better treatments are needed for CSU and several are currently under investigation (184), most of them mast cell-targeted (185, 186). These treatments aim to inhibit mast cell mediators, prevent mast cell activation (187), silence mast cells *via* inhibitory receptors, or deplete mast cells. One of the biggest challenges in treating CSU patients in the future will be to figure out which patients benefit best from which treatment. For example, fenebrutinib, an oral Bruton's tyrosine kinase inhibitor, has been shown to be most effective in type IIb autoimmune CSU (180). The identification of reliable and easy to analyze biomarker for response to treatment will thus be an important task for future research.

### Pemphigus

Pemphigus refers to a group of rare autoimmune blistering diseases characterized by autoantibodies targeting desmosomal cadherins: most commonly desmoglein-1 (Dsg1) and desmoglein-3 (Dsg3). It presents with localized or widespread flaccid bullae which can rupture and progress to post-bullous erosions and crusts (Figure 1K). There are two major types: Pemphigus vulgaris (PV) and pemphigus foliaceus (PF). These subtypes are differentiated by oral and/or mucous membrane involvement in PV, which is absent in PF. The histological hallmark of pemphigus is acantholysis, caused by loss of adhesion between effected keratinocytes (188). Overall, pemphigus is associated with significant morbidity and mortality (22, 189).

#### Diagnosis

The diagnosis can be made by direct immunofluorescence (IF) microscopy of a perilesional skin biopsy, revealing deposition of IgG autoantibodies and/or C3 on the cell surface of keratinocytes (190). Detection of antibodies against Dsg1 or Dsg3 using ELISA, or use of indirect immunofluorescence microscopy against monkey esophagus allows serologic characterization (188). Significant delays in diagnosis are unfortunately common (191). Barriers to obtaining direct immunofluorescence microscopy serve as a roadblock in the diagnosis of pemphigus, particularly in the developing world (192). Immunohistochemical approaches, and even desmoglein ELISA have significant sensitivity limitations furthering diagnostic delays when direct immunofluorescent microscopy is not feasible (193). In the so far largest multicenter prospective study, anti-Dsg1/ Dsg3 serum antibodies were, however, detected in 329 (98.5%) of 333 pemphigus sera diagnosed by the clinical picture and direct IF microscopy using widely available assays.

#### Treatment

The first-line treatment for pemphigus is systemic corticosteroids, often used in conjunction with other immunosuppressive agents (194). More recently, evidence suggests the use of the anti-CD20 monoclonal antibody rituximab as an alternative first-line agent used alongside corticosteroids (195, 196). Additional therapies such as intravenous immunoglobulin (IVIg) and immunoadsorption can be used as adjuvant treatments, either in combination with first-line medications or when contraindications are present (194). However, despite the significant advances in the treatment of pemphigus in recent years, there are still numerous limitations in current therapies. To achieve clinical response during the acute phase of disease, high dose corticosteroids are generally required (197). While novel treatments such as rituximab may reduce cumulative steroid dosages, they do not work quickly. Furthermore, relapses are also frequently encountered (198).

#### Perspectives

Thus, there is a need for short-term agents that can minimize the need for high dose steroids. Once achieving complete remission, relapses remain common, though this can be decreased with more aggressive protocols utilizing additional rituximab infusions (199–201). An alternative approach, for example targeting autoantibody-induced tissue pathology have emerged (202, 203). In addition, attempts to incorporate precision medicine into the treatment of pemphigus are on the horizon (19). However, optimism must be tempered by the contributory role of non-desmoglein autoantibodies in pemphigus and aberrant cell signaling, which contribute toward the pathogenesis (204–206).

### Bullous Pemphigoid

Bullous pemphigoid (BP) is one of the most common autoimmune blistering skin diseases, and it is characterized clinically by tense blisters with itchy urticarial erythema on the trunk and extremities (Figure 1L) (207). Mucosal surfaces can also be affected. It is most prevalent in the elderly (late 70s), but can appear in younger people (208). The molecules targeted by BP autoantibodies are the two hemidesmosomal proteins type XVII collagen (COL17, also called BP180) and BP230, and the former molecule has been recognized to be the major autoantigen. Triggering factors for BP include ultraviolet rays and other radiation, burns, trauma, and regulatory T-cell dysfunction (209–211). Dipeptidyl peptidase-4 (DPP-4) inhibitors have recently gained attention as a cause of BP (212–214).

#### Diagnosis

BP is diagnosed based on the clinical, histological, and immunological findings (215, 216). In addition to the clinical features of tense blisters and urticarial erythema and the histological feature of subepidermal blistering, the detection of tissue binding and/or circulating autoantibodies against the dermal-epidermal junction (DEJ) is essential. Direct IF microscopy of perilesional skin is the most sensitive method for detecting autoantibodies in BP, with a linear IgG and/or C3 deposition at the DEJ. To detect circulating autoantibodies, indirect IF microscopy using cryosections of normal human skin or 1M NaCl-split human skin is useful. To confirm the target antigen of autoantibodies, an ELISA using recombinant BP180 NC16A is widely used. A full-length BP180 ELISA (217) and a BP230 ELISA are also useful. Diagnostic challenges are infrequently encountered in patients presenting with “classical” BP lesions, i.e., tense blisters on erythematous skin. By contrast, atypical clinical presentations, which occur in least 20% of all BP patients, diagnosis is often delayed by several months, if not years (4, 218, 219).

#### Treatment

In clinically localized or mild cases, superpotent topical corticosteroids (clobetasol propionate) are applied to lesions only or to the whole body except the face as a first choice (215, 216, 220). Low-dose oral corticosteroids, tetracycline (and nicotinamide) and dapsone are also used. In generalized or moderate/severe cases, oral corticosteroids (0.5–1.0 mg/kg/day) or superpotent topical corticosteroids are the mainstay treatment. If sufficient efficacy cannot be achieved, immunosuppressants (e.g., azathioprine, mizoribine, cyclophosphamide, cyclosporin, mycophenolate mofetil, methotrexate), steroid pulse therapy, plasma exchange/immunonoadsorption, or intravenous immunoglobulins should be added as appropriate (215, 216, 221, 222). A randomized controlled trial demonstrated the efficacy of doxycycline (200 mg/day) as an initial treatment for BP. Non-inferiority was shown in comparison with oral prednisolone (0.5 mg/kg/day), and the safety was significantly higher (223). Whilst all these treatments, especially those using topical or systemic corticosteroids, induce remission in over 90% of the patients within 4 weeks, relapses during tapering corticosteroids or after stopping treatment are frequent (220, 222, 224). This necessitates prolonged treatment with corticosteroids. In turn, this long-term use of oral corticosteroids frequently causes severe side effects, particularly in the elderly. In addition, although most BP cases are well-controlled by standard therapies, intractable and recurrent cases still exist. Therefore, new treatments that can suppress the disease activity and reduce or replace (oral) corticosteroids are much anticipated.

#### Perspectives

Based on the clinical and immunological characteristics, some molecules are considered as promising targets for BP therapies. As in pemphigus, the anti-CD20 antibody rituximab has been reported as effective against BP (225, 226). The pathogenicity of IgE autoantibodies has been described in many studies (227–229), and the efficacy of the anti-IgE antibody omalizumab against BP has been reported (227, 230, 231). Furthermore, the anti-IL-4 receptor alpha dupilumab has been reported as an alternative to prednisolone (232, 233). Several clinical trials targeting these molecules are under way, which may provide new treatment options for BP in the near future (234). Regarding the early diagnosis, continued education of healthcare providers, especially outside dermatology, is important to raise awareness for (atypical) BP (219), as well as forms of drug-induced BP (24). One important pillar in raising the awareness for BP and other rare skin blistering autoimmune diseases is the International Pemphigus & Pemphigoid Foundation (IPPF), the largest patient organization for those affected by pemphigus or pemphigoid.

### Mucous Membrane Pemphigoid

Mucous membrane pemphigoid (MMP) is a subepithelial/ subepidermal blistering autoimmune disease with predominant involvement of orifice-close mucosal surfaces and autoantibodies against proteins of the dermal-epidermal junction (Figure 1M) (235). The main target antigens are BP180 (type XVII collagen) and laminin 332 recognized in about 80 and 10–20% of patients, respectively. In <5% of MMP patients, type VII collagen is targeted and individual patients with reactivity against a6b4 integrin have been described (207, 236). The incidence of MMP has been estimated to 1.3 and 2.0/ million/year in France and Germany (237–239) and its prevalence was calculated to be 24.6 patients/million, i.e., about 2,000 patients in Germany in 2014 (23) MMP mainly occurs between the age of 60–80 years and is extremely rare in children and adolescents (240, 241). The oral cavity and conjunctivae are the most frequently affected mucosal surfaces followed by nasopharynx and genitalia, and more rarely, larynx, esophagus, and trachea. In about 30% of patients, additional skin lesions may occur (241). MMP is associated with a considerable morbidity including pain, difficulties in food intake and breathing as well as visual impairments that can lead to blindness (241). Further studies are needed to assemble more data about the incidence and prevalence of MMP in different geographical regions. So far, epidemiological studies have been mostly limited to central Europe. In the Schleswig-Holstein registry of autoimmune blistering diseases including all newly diagnosed patients in the most northern German province (www.sh-register-pemphigoid-pemphigus.de) we are prospectively mining the annual incidences of MMP since 2016.

#### Diagnosis

For the management of MMP the recent S3 guidelines of the European Academy of Dermatology and Venereology will be instrumental (241, 242). Diagnosis of MMP is based on the presence of predominant mucosal lesions and the detection of tissue-bound and/or circulating autoantibodies (242). Direct IF microscopy of a biopsy taken form perilesional tissue or unaffected oral mucosa is the diagnostic gold standard with a sensitivity of 60–90% (241–243). Like in all pemphigoid disorders, in MMP, it reveals linear deposits of IgG, IgA, and/or C3 at the subepithelial basement membrane zone (BMZ). Repeated biopsies for direct IF can increase the sensitivity from 70 to 95% (243, 244). Indirect IF microscopy on human salt-split skin is a convenient and sensitive screening assay for circulating autoantibodies against the subepithelial BMZ and allows the differentiation between IgG/IgA that binds to the roof of the artificial split, i.e., antibodies against BP180, BP230, and a6b4 integrin and IgG/IgA that labels the blister floor as seen with reactivity against laminin 332 and type VII collagen (245–247). Widely available antigen-specific test systems include ELISA and/or indirect IF applying the recombinant NC16A domain of BP180 NC16A, the NC1-domain of type VII collagen, a C-terminal stretch of BP230, and the laminin 332 heterotrimer (242, 248–252). In particular, detection of anti-laminin 332 antibodies is essential since-anti-laminin 332 MMP is associated with a malignancy in 25–30% of patients. After the initial observation of solid malignancies in 2 of 5 MMP patients with serum autoantibodies against laminin 332 by Leverkus et al. (253), Egan et al. reported malignancies in 10 of 35 patients (29%) (254). This important clinical association has then been corroborated by several other studies (249, 253–259). In contrast, using an in-house ELISA Bernard et al., did not recognize the association of anti-laminin 332 IgG and malignancies (260). Recently, in a large multicenter study, a 6.8-fold higher risk of malignancy has been calculated in anti-laminin 332 MMP patients compared to the general population (237). However, serological diagnosis is limited by relatively low autoantibody levels. In addition, no standardized assay is widely available for serum IgG against the BP180 ectodomain outside the NC16A domain, an immunodominant stretch in anti-BP180 MMP. Since IgA reactivity is frequently seen in MMP, the lack of widely available test systems for IgA reactivity against BP180, BP230, and type VII collagen is further limiting the diagnostic power.

#### Treatment

The European S3 guidelines recommend the first-line use of topical corticosteroids with or without dapsone, methotrexate or tetracyclines for mild and moderate MMP and for severe MMP, dapsone in combination with systemic cyclophosphamide with or without systemic corticosteroids (242). However, apart from two small phase IIa trials comparing dapsone with cyclophosphamide and prednisone with cyclophosphamide, respectively, in MMP patients with ocular disease, no randomized control trials have been performed in MMP (261). Hence, well-designed clinical trials are urgently needed to identify the best current available treatment options for MMP patients.

#### Perspectives

The highly standardized indirect IF test based on the expression of recombinant laminin 332 in a human cell line has become widely available (249). This assay will be instrumental for the in-depth analysis of the occurrence of malignancies in patients with anti-laminin 332 MMP, an association that has not yet been widely recognized in the community. In this sense, the recommendation of the S3 guidelines to assay for anti-laminin 332 reactivity in all patients with negative or dermal binding by indirect IF microscopy on salt-split skin will propel our knowledge. For the management of anti-BP180 MMP only the anti-BP180 NC16A IgG ELISA is widely available. Since in MMP the NC16A domain is not an immunodominant region and IgA reactivity is frequently found, assays for the detection of serum IgA and IgG against other parts of the BP180 ectodomain are urgently needed. Considerable progress is being awaited on our understanding of the disease mechanisms in MMP using a recently established mouse model of anti-laminin 332 MMP (262). In contrast to the previously reported model by Lazarova et al. this model depends on Fc receptor-mediated inflammatory pathways and C5aR1 (262, 263). The future use of the novel model to preclinically evaluate future therapeutic strategies has recently been supported by the observation that dapsone, first-line treatment in MMP, resulted in a significant reduction of oral and cutaneous lesions compared to vehicle-treated mice (264). Nonetheless, until a mouse model for anti-BP180 MMP, that represents the large majority of MMP cases, has been developed, it will remain unclear whether the anti-laminin 332 MMP model fully represents experimental MMP. In any case, the present anti-laminin 332 MMP mouse model opens the possibility to pre-clinically test anti-inflammatory agents and as such pave the way for randomized controlled trials in MMP.

### Epidermolysis Bullosa Acquisita

Epidermolysis bullosa acquisita (EBA) is caused by autoantibodies targeting type VII collagen (COL7) which is a major component of anchoring fibrils (265, 266). Despite this singular key pathogenic principle, the clinical presentation of EBA is broad (Figures 1N,O). The disease may present as fragile skin with subsequent scaring, or as a widespread inflammatory disease with blistering and erosions. In addition to the skin and mucous membranes, internal organs may be affected (267). For example, strictures of the esophagus are relatively common (268). Thus, EBA imposes a high burden on the patients affected by this rare disease.

#### Diagnosis

EBA is confirmed if linear deposits of immunoglobulins and/or C3 are detected by direct immunofluorescence (IF) microscopy or perilesional skin biopsy and if an u-serrated pattern is seen in direct IF microscopy, or circulating COL7 autoantibodies are detected (247, 269). Due to the heterogeneous clinical presentation, EBA is often not considered as a differential. Thus, the challenge is to raise awareness for this rare disease because once considered as a differential, diagnosis can readily be obtained using direct IF microscopy and serology.

#### Treatment

There are no controlled clinical trials for EBA treatment which is thus based on expert recommendation. Unspecific immunosuppression is the mainstay of EBA treatment. Most commonly, systemic corticosteroids are used. In many cases additional immunosuppressants are added to systemic corticosteroids, most commonly azathioprine or cyclosporine are used (270). Overall, management of EBA is notoriously challenging—median time to remission is 9 months. In the same study, complete remissions were achieved in 45% of patients, with another 45% in partial remission and 10% with ongoing active disease−6 years after the initial diagnosis was made (271). Thus, treatments that induce remissions more reliably and faster are urgently needed to relieve the burden imposed by EBA.

#### Perspectives

In a metanalysis of over 1,000 EBA cases, use of the CD20 antibody rituximab or high dose intravenous immunoglobulin G (IVIG) were, compared to all other treatments, more often associated with the induction of remissions (270). These observations are a basis to establish protocols for clinical trials in EBA, evaluating the impact of either rituximab or IVIG. In addition, this also indicates that drugs targeting the B cells, such as the BTK inhibitor PRN1008, or compounds modulating the half-live of IgG, such as FcRn inhibitors, could be also effective in EBA (234). In addition, use of pre-clinical model systems has identified and validated a number of novel therapeutic targets in EBA (272–276). Based on these findings in pre-clinical EBA models, controlled clinical trials are currently performed—albeit in bullous pemphigoid patients (234).

## Autoinflammatory Diseases

### Cryopyrin-Associated Periodic Syndrome

Cryopyrin-associated periodic syndrome (CAPS) comprises a group of rare diseases that, despite certain clinical similarities, were previously considered separate disorders. These include Familial Cold Urticaria Syndrome (FCAS) which was first described in 1940 (277), Muckle-Wells Syndrome (MWS), and Chronic Infantile Neurologic Cutaneous and Articular or Neonatal Onset Multisystem Inflammatory Disease (CINCA/NOMID). Its prevalence is about 1–2 per million inhabitants in Europe and the USA.These diseases are characterized by an attack-like course with fever episodes lasting up to a few days and an enormous increase of inflammatory laboratory parameters, usually an accompanying urticaria-like exanthema, conjunctivitis, joint and muscle pain as well as hepatomegaly and splenomegaly (Figure 1P). In severe cases, cartilage growth leads to joint dysfunction. In MWS and NOMID/CINCA, central nervous involvement with mental retardation, epilepsy and hearing loss is found. The expression of the disease pattern varies from individual to individual. A long-term complication is the development of AA amyloidosis, which affects multiple organs, most prominently the heart and the kidney. As the cause for these diseases, heterozygous monogenetic deficiency in the NLRP3 (278), NLRP12 (279), PLCG2 (280), and NLRC4 (281) genes have been identified. However, a considerable number of cases are caused by mosaicism in the respective genes, such as in NLRP3 (282).

#### Diagnosis

The diagnosis is based on a careful history and observation of the clinical course, especially of the inflammatory parameters, as well as genetic testing, preferably requiring NGS panel diagnostics. It may be necessary to assess whether the disease can be influenced by corticosteroids, NSAIDs, and ultimately IL-1b inhibitors in an individual patient. The additional use of clinical scores, such as the EUROFEVER/PRINTO score (283), is helpful, although, given the rarity of the disease, a systematic approach is needed to differentiate it from other diseases. A special difficulty are cases where the disease is caused by somatic mosaicism or by a not yet identified unknown genetic defect. In such cases the diagnosis may not be made satisfyingly, leading to delay in efficient, but often expensive, treatment options. In addition to diagnosis, monitoring of disease activity is also of high importance. While ESR and CRP are routine diagnostics, the measurement of calprotectin levels or amyloid A in the serum, for example, are helpful but much less available. Another problem is the early diagnosis of AA amyloidosis and determination of its extent. Here, nuclear medicine methods such as a PET-CT scan with 18F-florbetaben have been described (284) but are also not yet routine.

#### Treatment

The IL-1β inhibitors anakinra and canakinumab are approved and available for the treatment of CAPS. However, other cytokines such as IL-18 have been described to be important in autoinflammatory diseases (285, 286). Hence, breakthrough attacks carried by IL-18 are not inhibited by current treatments. On the long run, secondary AA amyloidosis poses a challenge. Although it can be indirectly alleviated by inhibition of IL-1β signaling, targeted resolution of amyloid deposits is not possible to date (287).

#### Perspectives

Over the past 20 years, genetic defects have been identified for a variety of autoinflammatory diseases. Especially challenging are cases in which no classical germline mutation is present. In such cases, classical genetic methods reach their limits. The development of third generation sequencing methods such as nanopore sequencing with the simultaneous development of bioinformatics and advances in IT infrastructures could provide the solution for these cases as well (288). In addition to inhibition of secondary proinflammatory messengers such as IL-1β, inhibition of NLRP3 by small-molecule agents may also show promise (289, 290). To inhibit the action of IL-18, which is important in addition to IL-1β and plays a role in macrophage activation in particular, a promising drug might be available in the form of IL-18 binding protein (tadekinig alfa) (291).

### Schnitzler's Syndrome

Schnitzler's syndrome is a late-onset autoinflammatory diseases that has been described first in 1972 by Schnitzler (292). The disease is characterized by the combination of urticaria-like exanthema (neutrophilic urticarial exanthema) and gammopathy, associated with fever, joint, muscle or bone pain, elevated inflammation markers, morphologic bone changes, hepato-splenomegaly, and palpable lymph nodes (Figure 1Q) (29). It is considered to be a rare entity—with only about 100 patients described in the literature—although a retrospective database search for urticarial exanthema associated with dysproteinemia led to the identification of 16 patients at Mayo Clinic (293), pointing toward a much higher incidence. The etiology and pathogenesis of the disease is unknown. A somatic mutation is assumed (293), which is comparable to the pathogenesis of mastocytosis. In the case of mastocytosis, even low frequencies of mutant cKit that are barely detectable by means of digital PCR can lead to pronounced symptoms (294). The possible causal relationship between gammopathy and Schnitzler syndrome is also unclear.

#### Diagnosis

The diagnosis of Schnitzler's syndrome should be considered in patients with gammopathy and urticarial exanthema, especially those without itching, increased inflammation markers and fever (295). On the other hand, chronic spontaneous urticaria (CSU) is a much more common diagnosis, which renders the differentiation very difficult. Especially, pressure urticaria may present with systemic symptoms such as fever and myalgia (296). On the other hand, CSU is such a much more common disease than Schnitzler syndrome that not every gammopathy in combination with urticarial exanthema should be misdiagnosed as Schnitzler syndrome. Unfortunately, the diagnosis of chronic urticaria is not established by specific biomarkers that would allow differentiation from other entities including allergic forms. Nevertheless, a lack of response to antihistamines, biologics such as omalizumab, or even corticosteroids may serve as further evidence of an autoinflammatory syndrome. The Strasbourg criteria are helpful in establishing the diagnosis, although they are still considered provisional and specificity and sensitivity have not been adequately determined (297). The dermatohistopathological differentiation between neutrophil-rich infiltrates in Schnitzler syndrome (298), urticarial vasculitis, and urticaria is not straightforwardly possible in practical settings, although a morphologic criterion, neutrophilic epidermotropism, might be specific for autoinflammatory diseases such a Schnitzler's syndrome. Moreover, the gammopathy strictly required by the Strasbourg criteria may be not that absolutely necessary, as cases that appear to be clearly late-onset autoinflammatory diseases may present without it (299, 300). Gammopathy may also develop later in the course of the disease. Hence, establishment of validated diagnostic criteria for Schnitzler's syndrome is needed.

#### Treatment

IL-1β inhibiting treatment with anakinra and also canakinumab is highly efficient and can lead to resolution of symptoms within a few hours (301). Other treatment modalities such as colchicine, hydroxychloroquine, pefloxacin (29), and IL-6 inhibition with tocilizumab (302) are described. Like CAPS, Schnitzler's syndrome may lead to AA amyloidosis (303). AL amyloidosis due to gammopathy occurs, although rarely (304). Both types are difficult to treat, and no specific amyloid resolving treatment is known.

#### Perspectives

At the time being, only 9 controlled studies for Schnitzler's syndrome are listed in ClinicalTrials.gov, 5 of which that are using established IL-1β and IL-6 inhibitors have the status of being completed. A novel IL-1β inhibitor with affinity to IL-1α and IL-1Ra is recruiting, and a study that tests the histone deacetylase inhibitor ITF2357 (305) has an unknown status. Drugs that target the inflammasome may be able to prevent the activation of the autoinflammatory cascade (290). A pilot study using the NLPR3 inhibitor dapansutrile is listed as recruiting. Interestingly, a clinical observation of a resolution of Schnitzler's syndrome after haematopoietic stem cell transplantation may hint at the pathogenesis, e.g., a somatic mutation in bone marrow cells (306). This observation may also hint at a similar pathogenic pattern as in systemic mastocytosis, where myeloablative conditioning followed by (allogenic) stem-cell transplantation is used for treatment (307).

## Rheumatic Diseases

### Cutaneous Lupus Erythematosus

Lupus erythematosus (LE) is a multisystem autoimmune condition that ranges from skin to multiorgan involvement. While systemic lupus erythematosus (SLE) involves many systems, cutaneous lupus erythematosus (CLE) affects the skin and/or mucosal surfaces (Figures 1R,S). CLE can present with a variety of cutaneous manifestations and is accordingly subdivided into three major categories: acute CLE (ACLE), subacute CLE (SCLE), and chronic CLE (CCLE). Discoid lupus (DLE), a subset of CCLE, and SCLE are the most common forms of cutaneous lupus. Skin lesions are often a cause of significant disability and may be associated with underlying multisystem involvement secondary to SLE activity (308, 309). There are several pathways involved in the pathomechanism of CLE. Excess production of type I interferons (IFNs) has been implicated in the pathogenesis of SLE (29224681). Both plasmacytoid dendritic cells (pDCs) and cytotoxic CD8+T cells are known modulators of type 1 IFNs and seem to be critical in disease progression (310). Additionally, type I IFNs induce JAK/STAT signaling which are commonly upregulated in lesional skin (311).

#### Diagnosis

There are no standardized diagnostic criteria for CLE, though preliminary criteria have been developed for DLE (312). The diagnosis of CLE is largely based on clinical presentation, laboratory serologies, and histopathological findings (312). Hallmark cutaneous manifestations include malar erythema for ACLE, psoriasiform or annular lesions with central clearing for SCLE, and erythematous, scarring lesions for CCLE. Other clinical symptoms seen especially with DLE include scarring or non-scarring alopecia, scarring, and dyspigmentation. While these findings are suggestive, CLE is often misdiagnosed (313), especially as other autoimmune connective tissue diseases such as dermatomyositis (DM) (314). Diagnosis is supported with serologies demonstrating positive antinuclear antibody (ANA) or antibodies to double-stranded DNA (dsDNA), and anti-Smith (anti-Sm), but these are frequently absent. ANA is largely ubiquitous among rheumatologic conditions; only one-third of positive ANA serologies correspond with a diagnosis of LE (315). While a positive ANA is regarded as highly sensitive for SLE, there are numerous cases of ANA negative CLE with systemic findings that in the past would have been classified as SLE (316). Autoantibodies against dsDNA and Sm are more specific for SLE, though their median prevalence ranges from 30 to 70% (317, 318). Histopathological findings are used to aid in the diagnosis of CLE but are similarly not specific for CLE. Patterns such as interface dermatitis, dermal mucin deposition, and periadnexal lymphocytic infiltrates are present in both dermatomyositis and CLE. Even characteristic CLE findings on direct immunofluorescence (DIF), including granular immunoglobulin and complement deposition, are found in DM (314). Misdiagnosing CLE not only delays treatment resulting in more skin damage but prevents screening for potentially serious organ involvement.

#### Treatment

Since the approval of hydroxychloroquine in 1955, the Food and Drug Administration (FDA) has included three additional therapies for SLE: belimumab, a B-lymphocyte stimulator inhibitor, Anifrolumab, an anti-IFNAR receptor antibody, and voclosporin, a calcineurin inhibitor (319, 320). Belimumab and voclosporin are specifically approved for lupus nephritis. Currently, hydroxychloroquine is the only FDA-approved for CLE (313). Despite this, antimalarials [hydroxychloroquine (HCQ), chloroquine, and quinacrine] and topical corticosteroids remain first-line for the treatment of CLE. Topical calcineurin inhibitors may be used as an alternative to corticosteroids for sensitive areas of the skin and long-term use (313). About 65% of patients with CLE respond to some variation of these therapies (321). In CLE refractory to antimalarials, methotrexate (MTX), and mycophenolate mofetil (MMF) are the most effective immunosuppressives, but they may not be tolerated (322, 323). There are several reports of Azathioprine treating CLE, though MTX and MMF are typically more effective (324). Dapsone may be considered in recalcitrant CLE as there is some evidence of its success (325). Retinoids have demonstrated success in CLE as well, though long-term use is required, which increases the risk of adverse effects (326). Lenalidomide, a thalidomide analog, has recently been used for patients with refractory CLE (327). It shares similar efficacy to thalidomide with an improved safety profile (328). Though these therapies effectively reduce disease burden in patients, off-label use makes them difficult to obtain. For example, patients must pay out of pocket for quinacrine, a drug that has shown efficacy in patients that do not respond to HCQ alone (313). As there are no curative therapies for CLE, the medications listed above are intended only to mitigate disease burden. Even when properly managed, damage that developed due to previous disease activity is notoriously difficult to resolve.

#### Perspectives

Although only approved for SLE, Anifrolumab demonstrated improvements in cutaneous disease and may benefit those who meet criteria SLE with cutaneous involvement. There are several clinical trials measuring improvement of cutaneous disease in CLE as a primary outcome. As with Anifrolumab, these novel therapies frequently target the type I interferon pathway, identified as a leading driver of cutaneous lesions. One such monoclonal antibody, BIIB059, causes internalization of the blood dendritic cell antigen 2 receptor on plasma dendritic cells (PDCs), subsequently inhibiting type I interferons and other pro-inflammatory modulators. A phase 2 trial testing this therapy met its primary outcome, which measured improvement of cutaneous LE compared to placebo (329) and a phase 3 trial will begin shortly. VIB7734, another monoclonal antibody that targets PDCs, showed efficacy in CLE in a phase 1 trial (330) and is of interest for future trials in CLE, although an ongoing phase 2 trial is in SLE. Janus Kinase (JAK) inhibitors have demonstrated improvement in a range of dermatological conditions and are actively being investigated for SLE (331, 332). Further studies that include patients with moderate to severe skin disease are necessary to elucidate their potential benefit in CLE. Iberdomide, a potent thalidomide analog, recently demonstrated an impressive reduction in cutaneous activity in patients with SLE. Improvement in SCLE and a trend to improvement with DLE activity was seen, although those with ACLE did not show improvement (333). The variable response based on CLE subtype may highlight the need for subgroup analysis in future trials. It is important to note that CLE is difficult to classify, especially early in disease, and 20% of CLE patients have more than one subtype of CLE. As clinical trials continue to focus on cutaneous disease as a primary endpoint, new, well-supported therapies may be identified for use in CLE.

### Dermatomyositis

Dermatomyositis (DM) is thought to result from environmental triggers such as UV exposure, medications, infections, or malignancies in genetically predisposed individuals. Regarding genetic predisposition, mostly associations with HLA have been reported (334). Several characteristic cutaneous findings may be seen, including but not limited to symmetric macular erythema of the elbows, knees, or dorsal hands (Gottron's sign), papules of the dorsal metacarpophalangeal or interphalangeal joints (Gottron's papules), periorbital violaceous erythema (heliotrope rash), periungual telangiectasias, and macular erythema of the upper back (Shawl sign) and V-area of the upper chest (V-sign) (Figure 1T) (335). DM can also affect several other organ systems, potentially involving the skeletal muscle, lungs, heart, and esophagus. Especially interstitial lung disease is prevalent in almost 60% of DM patients (336). Thus, DM can have a large impact on patients' quality of life which tends to correlate with the amount of skin disease activity (337).

#### Diagnosis

The EULAR/ACR classification criteria for adult and juvenile idiopathic inflammatory myopathies (IIM) and their major subgroups, including DM, are the only validated DM classification criteria (338). Patients are scored based on age of symptom onset, presence of muscle weakness, skin manifestations, other clinical manifestations, laboratory testing, and muscle biopsy features and can be further subcategorized into a form of IIM, like DM, if their score meets the cut-off probability of 55% (338). Despite validated criteria and characteristic cutaneous features, many clinicians do not accurately diagnose DM patients as evidenced by a retrospective study that showed that 56% of DM patients referred to an academic medical center were incorrectly diagnosed, the majority of whom were labeled as lupus or undifferentiated connective tissue disease (339). Patients with no muscle involvement and at least 2 of 3 possible skin-related items (heliotrope rash, Gottron's papules, and Gottron's sign) can be classified by the EULAR/ACR criteria as having amyopathic DM (ADM), and a skin biopsy is encouraged in such patients (339). However, these criteria have limitations as at least one retrospective study showed that 26% of patients with confirmed ADM would not meet the EULAR/ACR classification criteria due to the specific cutaneous findings required (340). It is suggested that cancer-screening investigations should be undertaken once a diagnosis of DM is established due to the associated increased risk of malignancy (341). However, no evidence-based malignancy screening protocol for DM patients currently exists. Thorough characterization of the autoantibody response in DM patients is also important in this regard, as certain autoantibodies are associated with a high risk of cancer (342). In addition, a detailed characterization of the autoantibody response in DM also allows to differentiate organ involvement and prognosis (343). In summary, missing awareness, lack of definite diagnostic criteria and no evidence-based recommendation for cancer screening are diagnostic challenges in DM.

#### Treatment

Systemic corticosteroids, with or without immunosuppressives, are the mainstay of DM treatment when muscle disease is confirmed and often allows patients to improve their muscle symptoms (344). Many patients unfortunately have persistent cutaneous disease despite aggressive topical and systemic therapy. A common therapeutic ladder for the treatment of cutaneous DM involves antimalarials like hydroxychloroquine or chloroquine with or without quinacrine followed by methotrexate or mycophenolate mofetil and then IVIG, currently the only FDA-approved therapeutic for DM (345). However, even these more commonly used options have several limitations as DM patients have increased risk of cutaneous reactions to hydroxychloroquine than lupus patients, while steroid-sparing agents can have many side effects which are intolerable to patients (346). Very few randomized controlled trials have been performed in DM. However, other therapies with some evidence to support their use include topical corticosteroids, topical tacrolimus (347), topical pimecrolimus (348), tofacitinib (349), dapsone (350), and thalidomide (351), among others. One challenge facing the development of new therapies for DM is that clinical trials often use primary outcome measures which heavily weigh muscle involvement, like the TIS score. In the recently completed phase 3 study of lenabasum, the primary outcome was not met despite improvement in skin disease activity, highlighting the need for careful consideration of outcomes (352).

#### Perspectives

Much work is currently ongoing to overcome the diagnostic and therapeutic challenges facing DM. A recent international project that developed skin-focused classification criteria for DM that is more inclusive than the EULAR/ACR criteria while still excluding disease mimickers like lupus is undergoing prospective validation (353). While no consensus guidelines exist for cancer screening in DM patients, The International Myositis Assessment and Clinical Studies Group has an ongoing effort to create evidence-based malignancy screening guidelines for IIM patients (354). There is hope for improved therapeutic options for DM patients based on ongoing clinical trials as well as promising proof-of-concept studies. Additional therapeutics with trials that are ongoing or have reported data include JAK inhibitors, anti-interferon beta, subcutaneous immunoglobulin, and KZR-616 (349, 355).

### Systemic Sclerosis

Systemic sclerosis (SSc) is an autoimmune disease belonging to the connective tissue/rheumatic diseases, characterized by a triad of vasculopathy, inflammation, and fibrosis. SSc is a rare disease with a prevalence of 40–200:1,000,000 inhabitants (35). Clinically, SSc is characterized by a wide heterogeneity, ranging from skin findings to severe organ damage including gastrointestinal dysfunction, interstitial lung disease, pulmonary arterial hypertension, cardiac inflammation, arrhythmias, neurological deficits, or end-stage renal failure. Skin findings can include oedema, scleroderma as well as acral ulcers, necrosis, or gangrene (Figures 1U,V). In recent years, the understanding of pathomechanisms in SSc and concomitantly, the therapeutic options for the treatment of affected patients have improved, especially regarding pulmonary artery hypertension and interstitial lung diseases. This has led to a reduction in disease-related mortality (356). Nevertheless, a multinational study examining mortality in patients with SSc between 2005 and 2014 continued to demonstrate early patient death (357). In addition, health-related quality of life is significantly lower in patients with SSc compared to healthy controls (358) and compared to other autoimmune diseases (359). The involvement of the gastrointestinal tract, pulmonary arterial hypertension, Raynaud's phenomenon and digital ulcers represent disease manifestations that affect the quality of life of patients (360). In addition, symptoms such as pain, dyspnea or impaired hand function are frequently reported by the patients as determinants for the quality of life. In addition, symptoms such as erectile dysfunction, pruritus, psychological problems such as anxiety received insufficient consideration in diagnostics and the development of treatment strategies for patients. Therefore, despite significant improvements in the understanding of the disease and expansion of therapeutic options, there is still a high unmet medical need in SSc.

#### Diagnosis

Years before the development of disease-defining symptoms in SSc, a risk for disease development in the presence of Raynaud's phenomenon or puffy fingers can be predicted by determination of biomarkers such as antinuclear antibodies and changes in capillary microscopy (361). These changes are summarized in the concept of “early SSc.” Currently, patients at risk receive close clinical follow-ups. However, it is unclear whether and which early treatment would attenuate the course of the disease (362). Due to the heterogeneity of disease progression in SSc, the identification of biomarkers is central to predict the development and severity of organ manifestations, disease progression, and response to treatment. Although numerous biomarkers have been investigated in studies, only a few of these biomarkers have found their way into routine clinical practice (e.g., AT1R autoantibodies, ETAR autoantibodies) in specialized centers (363, 364). The identification of biomarkers for individual prediction of organ manifestations and severity of disease progression represents the basis for establishing the concept of individualized medicine in SSc.

#### Treatment

To date, drug treatment of patients follows a manifestation-based approach according to the EULAR recommendations (365). However, sufficient, evidence-based treatment strategies are lacking for several disease manifestations of SSc. One obstacle in the development of appropriate treatment options is that the pathophysiological mechanisms leading to specific disease manifestations are poorly understood to date, leaving only symptomatic treatment options available. These include exemplarily treatment of contractures, calcinosis cutis, acral necrosis, gastrointestinal involvement, fatigue, arthritis, or enthesitis. The highest agreement on treatment recommendations for patients with SSc is to consider immunosuppressive therapies particularly for the early inflammatory phase of the disease including autologeous stem cell transplantation. The use of angiotensin-converting enzyme inhibitors is recommended for scleroderma renal crisis. Prostacyclins, endothelin receptor blockers, phosphodiesterase-V inhibitors, and stimulators of soluble guanylate cyclase (sGC) were shown to be effective in the therapy of the obliterative vasculopathy. Here, combination therapies are increasingly applied particularly for the therapy of pulmonary arterial hypertension (PAH). Recently, nintedanib, a small molecule tyrosine-kinase inhibitor was approved for the therapy of SSc-associated lung fibrosis. Of note, the anti-CD20 antibody rituximab has recently been demonstrated to have beneficial effects on skin and lung fibrosis and seems to be effective also in PAH. It is approved in Japan for the treatment of SSc (366, 367). Since these disease manifestations affect most patients, studies are urgently needed to decipher pathophysiology and develop causal therapeutic approaches. Furthermore, medication adherence is poorly investigated. Only a few clinical studies address compliance of SSc patients. Improving drug adherence could increase remission rates and prevent secondary disease complications. Moreover, since there are options for the therapy of cardiac arrhythmias, such as pacemakers or defibrillators, the establishment of a structured assessment for corresponding diagnosing is required. In addition to the insufficiently investigated treatment options for organ-related disease manifestations, the global disease activity cannot yet be adequately controlled with the approved therapeutic agents in all patients. Due to the described heterogeneity of organ manifestations, an interprofessional and interdisciplinary team is necessary to achieve optimal management of individual disease manifestations. A survey of patients with SSc conducted in the USA with regard to their individual unmet medical needs revealed deficits with regard to the psychological care of patients (368). In many places, there is a lack of structures that ensure interdisciplinary treatment of patients, which also includes psychological co-care of patients. Besides psychological co-care patients require physiotherapy and physical therapy to attenuate contracture development. Therefore, a prioritized objective for the next few years should be to create awareness of the need for interdisciplinary treatment and to establish appropriate structures on this basis. While diagnostics and therapy often focus on organ manifestations leading to the high disease-associated mortality, affected patients often evaluate pain, fatigue as well as alleviation of Raynaud's phenomenon and gastrointestinal symptoms as a treatment priority (360, 369). Therefore, practitioners need to define the individual treatment goal with the patient, considering not only global health but also health-related quality of life.

#### Perspectives

Despite the progress made in deciphering the pathogenesis of SSc in recent years, the triggers of the disease and the mechanisms that lead to the heterogeneous disease manifestations and disease severity remain poorly understood. However, since an understanding of these mechanisms is the basis for the identification of key molecules in pathogenesis and thus new therapeutic options, deciphering the disease-driving mechanisms of SSc is urgently needed. A key requirement for this is the enrollment of patients in international registries as well as a close collaboration between patients, clinicians, and scientists.

## Perspectives

In conclusion, there are a multitude of challenges for the diagnosis and treatment of chronic skin inflammation. These are, however, different for each disease: At a generalized glance, for the common inflammatory skin diseases, especially psoriasis, atopic dermatitis and lichen planus, disease heterogenicity and the identification of biomarkers that allow to predict treatment responses are at the forefront of the medical needs. We assume that with the advent of more and more detailed molecular data from these patients, a stratification allowing personalized treatment options are on the horizon (63).

For the rare and orphan chronic skin inflammatory diseases, medical practitioners from all specialties need to be made aware of these diagnoses, for example pemphigus or dermatomyositis. Patient organizations, such as the International Pemphigus & Pemphigoid Foundation (IPPF), that educate patients and medical practitioners alike are key for this. This will then also lead to an earlier and more validated diagnosis, which are both essential to start the appropriate treatments. Regarding these, there is a high need to develop more selective, and potentially causal, treatments for chronic skin inflammation. The use of the chimeric antigen receptor (CAR)-T cell technology to selectively deplete autoreactive B cells in pre-clinical models of pemphigus is a milestone in reaching this goal (370).

These differences in unmet medical needs across the here discussed chronic, non-communicable inflammatory skin diseases, do, however, not allow to determine which of these diseases has the “most” or the “highest” unmet medical need. In a generalized manner, one could approach this open issue by a systematic and longitudinal assessment of patient reported outcomes across a wide range of chronic inflammatory skin diseases. This would allow to determine the burden of individual diseases at diagnosis, as well as at a time point where treatment should have had a positive impact on both objective and subjective disease symptoms.

Another challenge that is observed across almost all chronic (skin) inflammatory diseases is comorbidity. At the forefront of these are metabolic syndrome, (cardio)vascular and mental health diseases (348, 371–374). One hypothesis is that chronic skin inflammation drives the associated comorbidity (279, 375). By contrast, others provided evidence that the environment is a key driver for the observed comorbidity in chronic (skin) inflammation (376).

Thus, in perspective, we believe that we will observe significant changes how chronic skin inflammation is diagnosed and treated during the next years. Overall, this will improve the quality of life of patients. We also envision the emerge of curative treatments for those autoimmune skin diseases, where culprit cells can specifically be targeted, i.e., autoreactive B cells.

## Author Contributions

RL: conceptualization. KBi (lead), HU, DR, MS, SS, MMe, MMa, DT, ES, CC, KA, DD, MH, AR, HG, AH, AS, GR, GS, JD, DZ, TS, AC, KW, RS, KK, VW, and RL: visualization. All authors: writing–original draft and review and editing. All authors contributed to the article and approved the submitted version.

## Funding

This work has been financially supported by the Cluster of Excellence Precision Medicine in Chronic Inflammation (EXC 2167) and the Collaborative Research Center Pathomechanisms of Antibody-mediated Autoimmunity (SFB 1526), the Research Units PruSearch (FOR 2690) and PEGASUS (FOR 2497), the Individual Research Grant RI/1056-11, all from the Deutsche Forschungsgemeinschaft; the Schleswig-Holstein Excellence-Chair Program from the State of Schleswig Holstein, an EADV research fellowship grant 2019, the Department of Veterans Affairs Veterans Health Administration, Office of Research and Development, Biomedical Laboratory Research and Development, National Institutes of Health (National Institute of Arthritis and Musculoskeletal and Skin Diseases) R01AR071653 (VPW) and R01AR076766 (VPW), and Sinergia Unravel principles of self-organization in injured tissue (CRSII5_202301/1) from the Swiss National Science Foundation.

## Conflict of Interest

HU has received research grants from JB, Otsuka, Taiho, Boehringer Ingelheim, Kyowa Kirin, Kaken, Sun Pharma, Shionogi, Teijin, Mitsubishi Tanabe, Nihon Zoki, Eisai, Torii and Tokiwa, consultant fees from Ono, Nihon-Pharmaceutical, Sun Pharma, argenx and Ishin Pharma, and speaker's fees from Nihon-Pharmaceutical, Maruho, Eli Lilly, Abbie, Eisai, Sanofi, Janssen, Kyowa Kirin, Ono, UCB, Novartis, Sun Pharma, Torii, Taiho, Mitsubishi Tanabe, and Boehringer Ingelheim during the last 3 years, DR has received honoraria or research support from AbbVie, Abcuro, AltruBio, Amgen, Boehringer-Ingelheim, Bristol Meyers Squibb, Celgene, Concert, CSL Behring, Dermavant, Dermira, Galdrema, Incyte, Janssen, Kyowa Kirin, Lilly, Merck, Novartis, Pfizer, Regeneron, Sanofi, Sun Pharmaceuticals, UCB, and VielaBio during the last 3 years, MS has received consulting/speakers' fees, or grant support from AbbVie, Almirall, Amgen, Biogen, BMS, Janssen, Novartis, and UCB during the last 3 years, SS has received funding and personal fees from Celldex, Clexio, Dermasence, Galderma, GSK, Kiniksa, Menlo, Trevi, Novartis, Sanofi (investigator all), Abbvie, Almirall, Beiersdorf, Bellus Health, Benevolent, Bionorica, Cara, Celgene, CelloHealth, Clexio, DS Biopharma, Eli Lilly, Escient, Galderma, Grünenthal, Kiniksa, Klinge Pharma, Menlo, Sanofi, Sienna, Trevi, P.G. Unna Academy, Perrigo, Pfizer, Vanda, Vifor, WebMD (Consultancy/Advisory board) and Almirall, Eli Lilly, Sanofi, Galderma, Menlo, Omnicuris, Beiersdorf, Leo Pharma, Novartis, P. G. Unna Academy, Pfizer, Pierre Fabre (speaker) during the last 3 years, MMe has received honoraria as a speaker and/or consultant for Amgen, Aralez, argenx, Bayer, Beiersdorf, Celgene, Escient, Galderma, GSK, Menlo, Moxie, Novartis, Pharvaris, Pfizer, Roche, Sanofi, Siennabio, and Uriach, MMa was a speaker and/or advisor for and/or has received research funding from Allakos, Amgen, Aralez, ArgenX, AstraZeneca, Celldex, Centogene, CSL Behring, FAES, Genentech, GIInnovation, Innate Pharma, Kyowa Kirin, Leo Pharma, Lilly, Menarini, Moxie, Novartis, Roche, Sanofi/Regeneron, Third HarmonicBio, UCB, and Uriach during the last 3 years, is a consultant, advisory board member, and/or investigator for AbbVie, Almirall, Amgen, Beiersdorf, BMS, Boehringer Ingelheim, Eli Lilly, Galapagos, Janssen-Cilag, LEO Pharma, MorphoSys, Novartis, Pfizer, Regeneron Pharmaceuticals, Inc., Samsung, Sandoz, Sanofi, Sun Pharma, and UCB, ES has received research grants from UCB, Incyte, Biotest, ArgenX, Dompe, Fresenius Medical Care, Bayer, AstraZenca, and Euroimmun and honoraria from Biotest, Thermo Fisher, ArgenX, Fresenius Medical care, Topas, Leo, Chugai, AstraZenca, and Almirall during the last 3 years, MH has received honoraria from Novartis, Sanofi, Celgene, and unrestricted grants from Biotest, Janssen Cilag and Topas durimg the last 3 years, KBi has received research funds from ArgenX during the last 3 years, DZ has received support for research and development work, lecturing and consulting from Euroimmun AG, UCB Pharma, ArgenX, Biotest, Abbvie, Janssen, Sanofi in the last 3 years, KW has received research grants, travel grants, consulting honoraria or lecturer's honoraria from Amgen, Bristol Myers Squibb, Celgene, Charité Research Organization, Flexopharm, Janssen-Cilag, Novartis Pharma, Sanofi-Aventis, and Trial Form Support during the last 3 years, RS has received research grants or honoraria for participation in advisory boards, clinical trials, or as speaker for one or more of the following: AbbVie, Amgen, Bayer, Boehringer Ingelheim Pharma, Bristol Myers Squibb, Celgene, Charité Research Organization, CSL Behring, Dr. Willmar Schwabe, Flexopharm, Incyte, Janssen-Cilag, La Roche-Posay Laboratoire Dermatologique, Novartis Pharma, Parexel International, Sanofi–Aventis, TFS, and UCB Biopharma during the last 3 years, VW has received grants from Celgene, Amgen, Janssen, Biogen, Gilead, Viela; Horizon therapeutics, Pfizer, Corbus, CSL Behring, consulted Astra-Zeneca, Pfizer, Biogen, Celgene, Resolve, Janssen, Gilead, Lilly, BMS, Nektar, Abbvie, Viela, GSK, EMD Serona, Sanofi, Anaptysbio, Amgen, Merck, Pfizer, Janssen, Neovacs, Idera, Octapharma, CSL Behring, Corbus, Novartis, Romefor during the last 3 years, RL has received honoraria for speaking or consulting or has obtained research grants from Novartis, Lilly, Bayer, Dompe, Synthon, Argen-X, and Incyte during the last 3 years. The remaining authors declare that the research was conducted in the absence of any commercial or financial relationships that could be construed as a potential conflict of interest.

## Publisher's Note

All claims expressed in this article are solely those of the authors and do not necessarily represent those of their affiliated organizations, or those of the publisher, the editors and the reviewers. Any product that may be evaluated in this article, or claim that may be made by its manufacturer, is not guaranteed or endorsed by the publisher.
